# CNT Network Impacts on Electrolyte-Gated Carbon Nanotube Field-Effect Transistors pH Sensors

**DOI:** 10.3390/s26165100

**Published:** 2026-08-12

**Authors:** Alireza Zare, Danica Fontein, Colm Carraher, Natalie O. V. Plank

**Affiliations:** 1 School of Chemical and Physical Sciences, Victoria University of Wellington, Wellington 6021, New Zealand; alireza.zare@vuw.ac.nz (A.Z.); danica.fontein@vuw.ac.nz (D.F.); 2 The MacDiarmid Institute for Advanced Materials and Nanotechnology, Wellington 6021, New Zealand; 3 Scentian Bio Ltd., Auckland 1025, New Zealand; colm@scentianbio.com

**Keywords:** carbon nanotube field-effect transistor, electrolyte-gated CNT-FET, pH sensing, CNT-FET electronic properties

## Abstract

Carbon nanotube field-effect transistors (CNT-FETs) are promising platforms for electrolyte-gated sensing, although the influence of CNT network density on device performance for pH measurements remains unknown. In this work, CNT-FETs with controlled network densities were fabricated from aqueous CNT solutions by varying the CNT concentration and the deposition time. Increasing CNT density improved conductivity, reducing channel resistance from the GΩ range to ~100 kΩ and increasing the on-current of electrolyte-gated devices from ~10 nA to ~1 µA. The fabricated CNT-FETs operating in a liquid-gated configuration exhibited on/off ratios ranging between 10^3^ and 10^5^. The minimum subthreshold swing achieved by the CNT-FETs was 77.5 mV/dec for devices employing a low-density CNT network, compared with 105 mV/dec for those incorporating a high-density CNT network. The high-density CNT networks also exhibited reduced electrostatic gate coupling due to charge screening effects. pH measurements in 1XPBS showed that low-density networks achieved the highest sensitivity of 8.9%/pH, whereas high-density networks showed lower sensitivity of 2.1%/pH, despite producing the largest absolute current response of ~70 nA. Medium-density networks provided the best balance between sensitivity, noise, and signal stability. These findings demonstrate that CNT network density is a critical parameter for optimizing electrolyte-gated CNT-FET pH sensors.

## 1. Introduction

Carbon nanotubes (CNTs) have emerged as one of the most promising nanomaterials for electronic applications because of their unique one-dimensional structure and exceptional electrical properties [1,2,3,4]. The field began with the discovery of CNTs by Sumio Iijima in 1991 [5]. Tans et al. demonstrated the first carbon nanotube field-effect transistor (CNT-FET), establishing CNTs as molecular-scale transistors capable of functioning as switching devices in electronic industries [6]. Later studies further advanced CNT-FET technology by introducing electrochemical and electrolyte gating approaches. M. Krüger et al. were the first to investigate the use of an electrolyte as the gate instead of a conventional back gate in a multiwall CNT-FET and reported enhanced gate capacitance and tuning between p-type and n-type conduction of a single CNT channel through Fermi-level shifting [7]. Rosenblatt et al. reported excellent subthreshold swing of ~80 mV/decade, close to the theoretical limit in a single-wall CNT-FET [8]. These developments also revealed that the electrical properties of CNTs are highly sensitive to their surrounding environment, where interactions with molecules, ions, and surface charges can alter their electrical behaviour, making them suitable for sensing applications.

Optimizing CNT-FETs for sensing applications requires a fundamental understanding of the mechanisms responsible for signal generation. Consequently, significant research efforts have focused on understanding how interactions between charged species in the electrolyte and carbon nanotubes influence the electrical response of CNT-FET devices. Early work by Chen et al. showed that sensing signals mainly arose from protein adsorption affecting metal-CNT contacts rather than the CNT channel itself, emphasizing the importance of contact-induced electronic effects [9]. Subsequently, Artyukhin et al. showed that electrostatic gating is a dominant sensing mechanism, where adsorbed charged molecules shift transistor threshold voltages through local electric fields [10]. Later, Heller et al. refined this understanding by experimentally distinguishing between several proposed mechanisms, including electrostatic gating, Schottky barrier modulation, mobility changes, and gate-coupling effects [11,12]. Their findings showed that sensing responses are primarily governed by electrostatic gating and Schottky barrier effects, with electrostatic gating being the more reproducible mechanism, particularly when the contact regions are passivated.

The first CNT-based chemical sensor was demonstrated by Kong et al., showing that adsorption of gas molecules such as NO_2_ and NH_3_ caused significant changes in nanotube conductance [13]. pH sensing has also been widely investigated. Early studies demonstrated that CNT-based devices could detect pH through changes in conductance and field-effect behaviour, establishing CNTs as promising ultrasensitive sensing platforms [14]. Back and Shim [15] further investigated the intrinsic pH response of liquid-gated individual single-walled carbon nanotube transistors and reported a systematic shift in the threshold voltage with increasing and decreasing pH. Heller et al. [16] demonstrated that pH changes alter the transfer characteristics of electrolyte-gated CNT-FETs and showed that the overall response arises from multiple mechanisms, including electrostatic gating, Schottky barrier modulation, gate-capacitance changes, and ionizable surface groups on the CNT surface and surrounding interfaces. Haeberle et al. [17] investigated solution-processed random CNT network transistors operated in electrolytes of different pH and reported a pH sensitivity of 19 mV/pH.

Later, CNT-based architectures such as extended-gate FETs [18,19], chemiresistors [20,21] and flexible thin-film devices were developed [22,23]. Recent advances in functionalization strategies and device architecture have further enhanced sensing performance, achieving wide pH detection ranges, and high sensitivities approaching or exceeding the Nernst limit [24,25]. Across CNT-FET-based pH sensing studies, carbon nanotubes contribute directly to the sensing mechanism, where pH-induced surface charge variations electrostatically modulate the CNT channel conductivity. Consequently, electrostatic gating of the CNT network is the fundamental mechanism underlying signal transduction in CNT-FET-based pH sensors [17]. Despite investigations into enhancing CNT-FET-based pH sensor’s sensitivity [24,26], and stability [27], by different approaches such as CNT surface treatment [28], surface passivation [27], or functionalization [24] of the CNTs, the influence of CNT network density which directly impacts the core underlying pH sensing mechanism in CNT-FET-based pH sensors remains insufficiently understood. Consequently, CNT network density can alter charge transport pathways, CNT-CNT junction resistance [29,30], electrostatic screening [11,31], and interfacial electrostatic coupling [32], which may affect both device characteristics and pH sensing performance. Therefore, this study investigates the effect of CNT network density on the electrical characteristics and pH sensing mechanisms of liquid-gated CNT-FET devices. pH sensing was employed as a model platform to evaluate CNT-FETs with different network densities and to examine how CNT network variations influence electrostatic gating effects and sensing response. Our results indicate a nearly 7%/pH increase in sensor sensitivity and 40% increase in normalized current by utilizing a low-density CNT network film. This work provides a clear understanding of the relationship between CNT network morphology and sensing mechanisms, contributing to the design and optimization of liquid-gated CNT-FET platforms for pH sensing and biosensing applications.

## 2. Experimental

### 2.1. CNT Network Film Fabrication

Alignment markers were patterned onto Si wafers with a 300 nm thermally grown SiO_2_ layer (WaferPro LLC, San Jose, CA, USA) via a standard photolithography process. The AZ1518 (MicroChemicals GmbH, Ulm, Germany) photoresist was deposited onto the Si/SiO_2_ wafer via spin-coating at a speed of 4000 rpm for 1 min, using a spin coater (WS-650-23, Laurell Technologies Corporation, PA, USA). The deposited layer of photoresist was then baked on a hotplate at 95 °C for 2 min. A MJB3 mask aligner (Karl Süss GmbH, Munich, Germany) was used to expose the photoresist-coated Si/SiO_2_ wafers to UV light with a wavelength of 365 nm and an intensity of 15 (mj^−1^cm^−2^) for 15 s. The UV-exposed wafers were soaked in AZ 726 MIF (MicroChemicals GmbH, Ulm, Germany) developer for 15 s. The metallization of the alignment markers with 10/60 nm Cr/Au was performed via thermal evaporation using an NEX DEP 200 evaporator (Angstrom Engineering Inc., Cambridge, ON, Canada), prior to lift-off in acetone. The Si/SiO_2_ wafers with alignment markers were cut into 12 mm × 12 mm chips then rinsed with DI water, acetone and IPA and N_2_ dried.

A hydrophilic surface on the Si/SiO_2_ chips was then created via an oxygen plasma run with 50 W power for 10 min using a PE-50XL Plasma cleaner (Plasma Etch Inc., Carson City, NV, USA). The Si/SiO_2_ chips were coated with Poly-l-Lysine (PLL) (0.1% (*w*/*v*), Sigma-Aldrich, St. Louis, MO, USA) solution via drop casting on the chip to cover the entire surface. The coating was left in contact with the substrate surface for 10 min, prior to rinsing with DI water and drying with N_2_.

**Figure 1:**
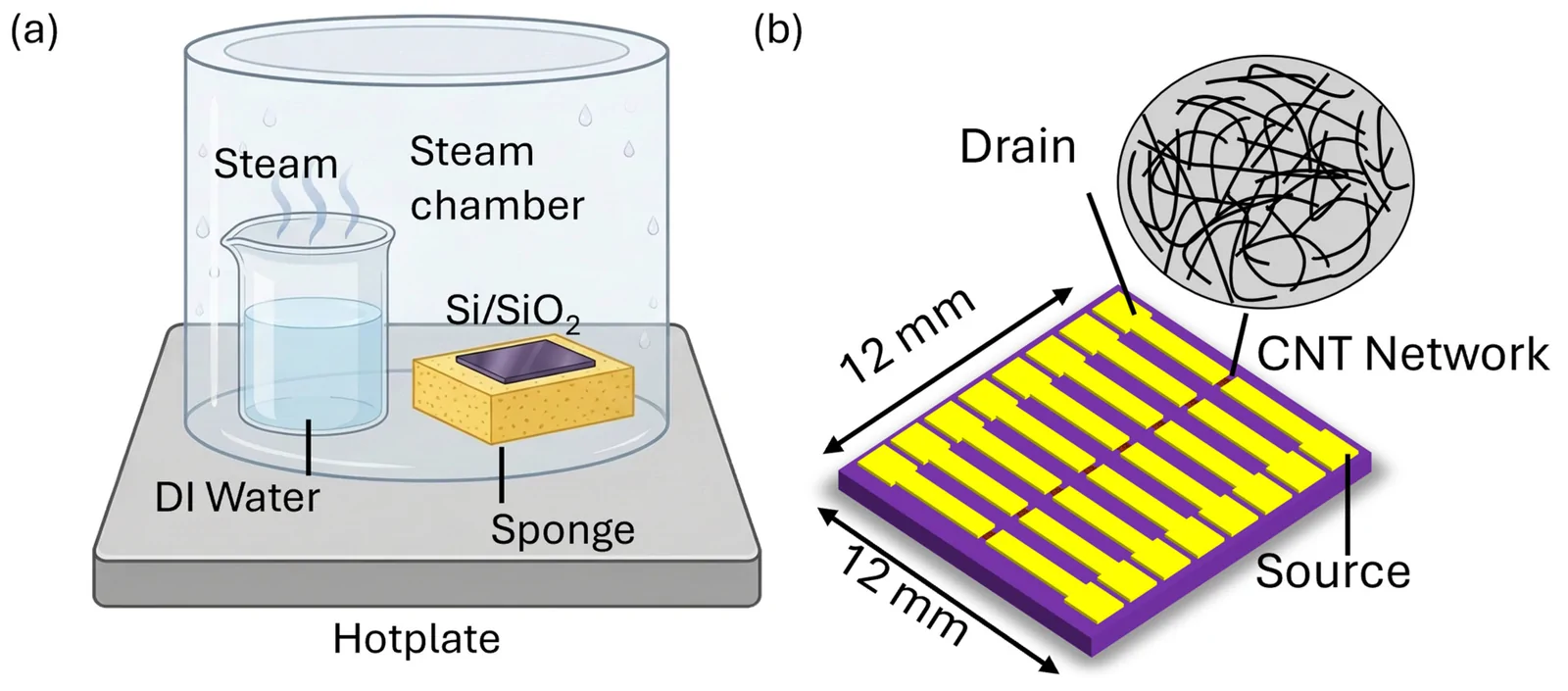
(**a**) Schematic of steam-assisted carbon nanotube network deposition setup. (**b**) Schematic of fabricated device with 8 individual CNT-FETs on a chip.

IsoNanotube-S 95% (NanoIntegris Technologies Inc., Boisbriand, QC, Canada) semiconducting single-walled carbon nanotube dispersions were deposited using four CNT concentrations of 100%, 80%, 50%, and 30% of the as-received dispersion and five deposition times of 1, 3, 5, 15, and 20 min. The as-received aqueous dispersion consisted of deionized water, high-purity single-walled CNTs, and a proprietary ionic surfactant mixture. The lower than 100% CNT concentrations were prepared by diluting the original dispersion with the corresponding nanotube-free surfactant solution supplied with the product. This approach maintained a constant surfactant composition while varying only the CNT concentration. A steam-assisted deposition setup adapted from the method reported by Begzaad et al. was used for depositing CNTs [33], as shown schematically in Figure 1a. The Si/SiO_2_ chips were placed directly on top of a sponge on top of a hotplate. The role of the sponge was to avoid the direct heat from the hotplate evaporating the solution too quickly. A beaker filled with DI water was used as a source of steam with a larger beaker placed on top of the setup as a chamber to keep the steam inside. The chips were then removed from the hotplate and thoroughly rinsed with DI water after the specified deposition times to fully wash away the excess CNT solution, then dried with N_2_. The CNT films were annealed at 150 °C for 1 h to remove the excess surfactant solution from the CNT sidewalls. The random network films of CNTs were then defined into isolated channels on the device chip via photolithography. The same photolithography process used for alignment markers was carried out to pattern the CNT network channels on the chip. An oxygen reactive ion etcher (Plasma lab 80 Plus, Oxford Instruments plc, Oxfordshire, UK) was used to etch the undesired CNT network film at 200 W and 600 mTorr for 3 min, leaving behind the 8 random network CNT channels with dimensions of 200 × 20 µm on the chip ready for electrode metallization.

### 2.2. Drain–Source Metallization

To define electrodes on the chip the negative photoresist AZnLOF 2020 (MicroChemicals GmbH, Ulm, Germany) was deposited on the chips over the patterned CNTs, via spin coating at a speed of 3000 RPM for 1 min. The MJB3 mask aligner was used to align the electrode mask and expose the chips to the UV light for 5 s. The chips were soaked in AZ 326 MIF (MicroChemicals GmbH, Ulm, Germany) developer for 80 s to define the electrodes. The fabrication of the drain and source electrodes was then performed by depositing 10 nm chromium followed by the deposition of a 60 nm gold film. The lift-off process was then performed by soaking the chips in acetone overnight. AZ1518 photoresist was spin-coated on the chip and hard-baked at 200 °C for 10 min followed by an oven bake at 150 °C for an hour, making the resist cross-linked to form a 1.2 µm thick encapsulation layer on the source and drain electrodes. This process left an open region in the CNT-FET channel with dimensions of 100 μm in length and 10 μm in width. Figure 1b shows the schematic of a fabricated device with 8 individual CNT-FETs on a chip after electrode metallization.

## 3. Characterization

### 3.1. Atomic Force Microscopy

Atomic force microscopy (AFM) scans were acquired in a dynamic force mode using Nanosurf Naio. The Nanosurf Naio (version 3.8) software imaging wizard was utilized to configure the settings. The anticipated feature height was 10 nm. The imaging process involved capturing images at a moderate scan speed with a resolution of 512 points per line. AFM scans were analyzed with Gwyddion (version 2.71) software.

### 3.2. Electrical Characterization of CNT-FET Device

Electrical characterization of the CNT-FET devices was carried out using a Keysight B1500A Semiconductor Device Parameter Analyzer (Keysight Technologies, Santa Rosa, CA, USA). Two-terminal current–voltage (IV) measurements were performed to evaluate the electrical transport properties of the CNT networks. The drain–source voltage was swept forward and reverse between −1 V and +1 V for all devices, and the corresponding drain–source current was recorded. The voltage sweep was conducted at room temperature under ambient conditions using a probe station and probe needles. The resistance of the CNT network was extracted from the linear region of the IV curve.

**Figure 2:**
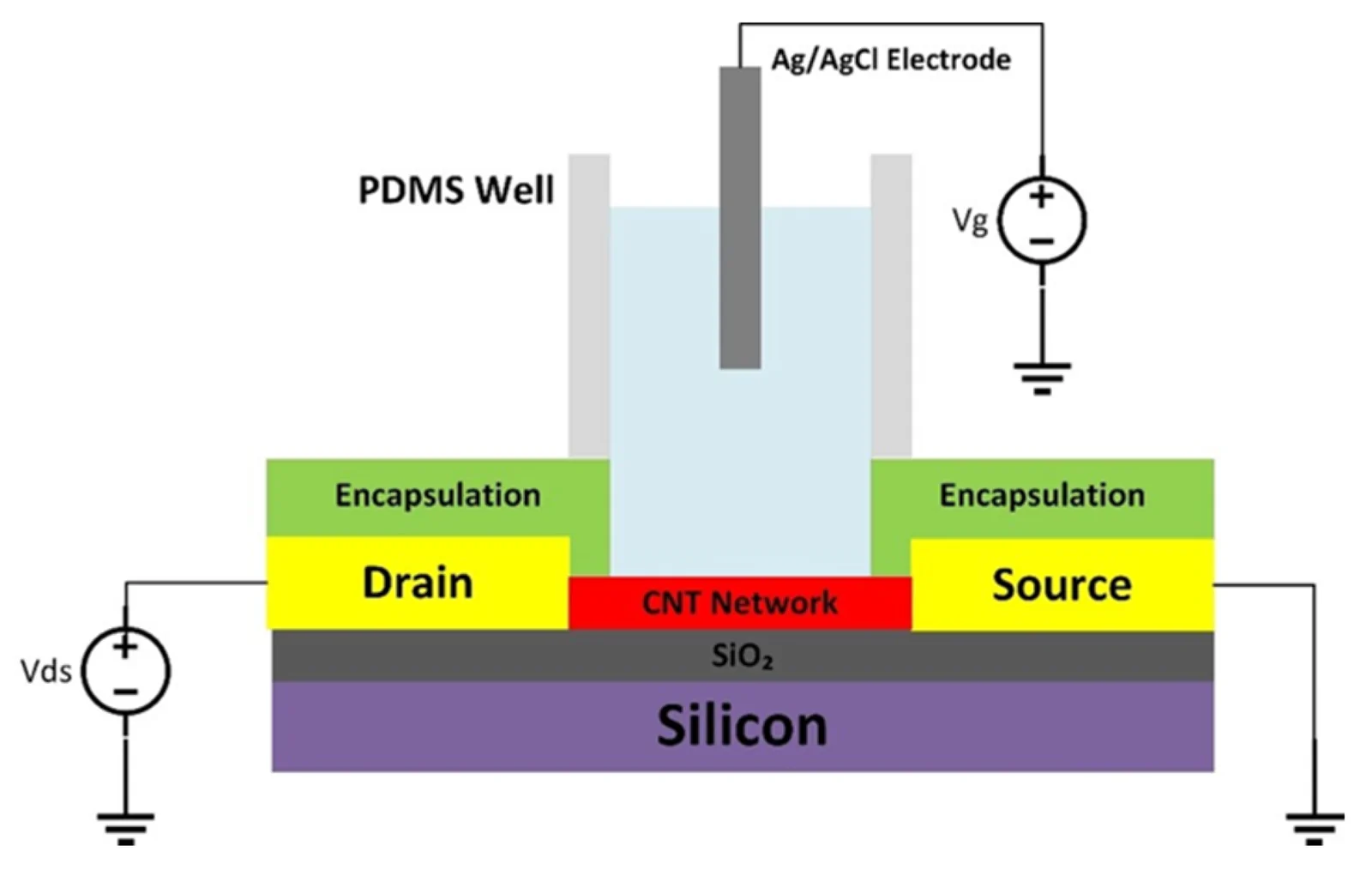
Schematic of liquid-gated CNT-FET device.

To carry out the transfer characteristics and pH measurement experiments, a polydimethylsiloxane (PDMS) (Sylgard 184 Elastomer Kit, Dow Inc., Midland, MI, USA) well was used to confine electrolyte solution (1 × phosphate-buffered saline pH 7.4 (1XPBS)) over the CNT channel, with an Ag/AgCl reference electrode serving as the gate electrode. For transfer curve sweeps the source and drain electrodes were contacted using probe needles from Micromanipulators. A constant voltage of 100 mV was applied to the drain and source electrodes with gate voltage sweeping forward and reverse between −0.5 and 1 V while the current was measured using the Keysight B1500A Semiconductor Device Parameter Analyzer. For the pH experiments in this study, an in-house multiplexing system consisting of a relay switching board controlled by a Raspberry Pi 4 and custom Python script (Python 3.12) was integrated with the SMUs of the Keysight B1500A Semiconductor Device Parameter Analyzer and a Keysight B2201A low-leakage mainframe (Keysight Technologies, Santa Rosa, CA, USA) to enable automated electrical characterization of device channels. Electrical contact to the device electrodes was established using pogo pins connected to the multiplexing board. A constant voltage of 100 mV for the drain and source and 0 V for the gate was applied while the real-time current was measured by the multiplex system. Figure 2 depicts the cross-sectional view of liquid-gated CNT-FET device used in this study.

### 3.3. pH Sensing Method

For the pH measurements, PBS solutions with pH values of 5, 6, 7.4, 8, and 9.5 were prepared. A 10X PBS stock solution was first prepared using a phosphate-buffered saline tablet (PBS) (Sigma-Aldrich, St. Louis, MO, USA). The desired pH solution was then adjusted using 0.1 mM HCl or NaOH and verified using a PC 2700 pH metre (Eutech Instrument Pte Ltd., Singapore). The solution was subsequently diluted with DI water while monitoring the pH to obtain the final 1X PBS buffer. To minimize potential variations in pH arising from temperature fluctuations, all electrical measurements were performed in a cleanroom laboratory with a humidity- and temperature-controlled environment (19–21 °C). During sensing experiments, 100 µL of pH 5 buffer was initially applied into the PDMS well containing the CNT-FET device. Subsequently, 50 µL aliquots of pH 6, 7.4, 8, and 9.5 buffer solutions were sequentially introduced every 300 s. The pH 9.5 measurement was incorporated during the later stages of the study to extend the sensing range. Consequently, some earlier measurements include pH values only up to pH 8, whereas all other experimental conditions remained identical.

## 4. Results and Discussion

**Figure 3:**
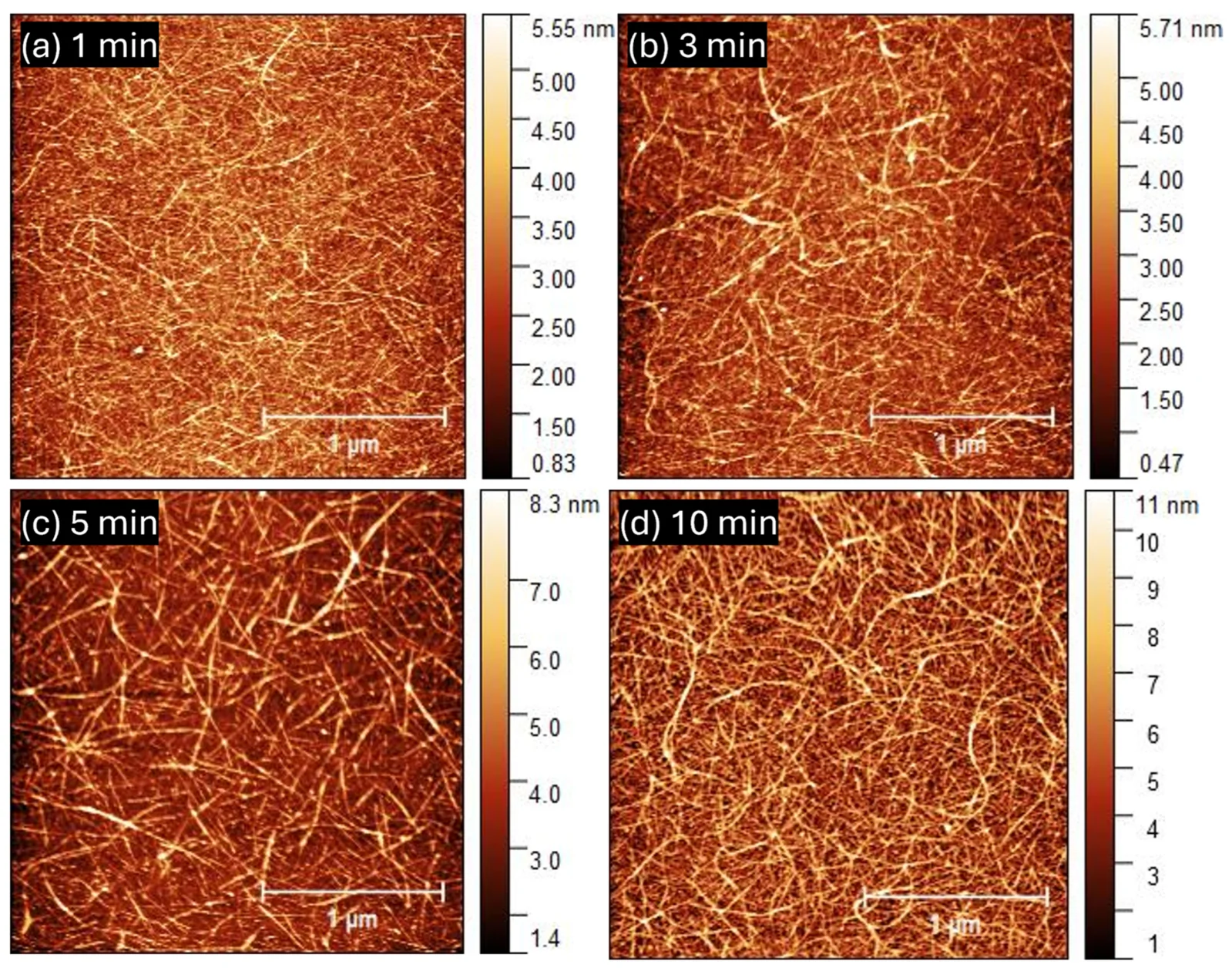
AFM topography images of CNT networks deposited from a 100% CNT solution with increasing deposition time: (**a**) 1 min, (**b**) 3 min, (**c**) 5 min, and (**d**) 10 min. The images show a progressive increase in CNT surface coverage and junction density with deposition time, indicating higher CNT network density and improved connectivity. Scale bar: 1 µm.

Atomic force microscopy was used to examine the morphological evolution of the CNT networks as a function of deposition time. Representative AFM images for CNT films deposited for 1, 3, 5, and 10 min with 100% CNT concentration are shown in Figure 3. At short deposition times (1 min), the CNT network is sparse, with limited nanotube coverage and relatively few inter-tube junctions, indicating a low-density network, depicted in Figure 3a. As the deposition time increases from 3 min, Figure 3b, to 5 min, Figure 3c, the surface coverage of nanotubes and the density of CNT-CNT junctions progressively increase, forming a more interconnected network. For the longest deposition time of 10 min, Figure 3d, the CNT network forms a highly dense mesh with numerous overlapping layers of nanotubes.

To quantitatively evaluate the evolution of CNT network connectivity with deposition time, an automated AFM image analysis method was employed to determine the junction density and effective segment density of the films [33]. A detailed description of the quantitative CNT network analysis is included in the Supporting Information. CNT network analysis was performed on the 50% and 30% diluted CNT network films because their sparse, predominantly single-layer morphology allowed individual CNTs, junctions, and conductive pathways to be clearly identified and quantified. In contrast, the dense multilayer structure of the 100% and 80% CNT films resulted in extensive CNT overlap, making the reliable determination of junction density and segment connectivity impractical. Representative AFM images and corresponding quantitative network analysis results for 50% and 30% CNT network films are shown in Figures S1–S4. For both the 50% and 30% CNT networks, increasing the deposition time resulted in progressively higher CNT surface coverage, leading to significant increases in both junction and segment density as the CNT networks evolved from sparse structures containing isolated nanotubes to dense, highly interconnected networks. For the 50% CNT network, increasing the deposition time from 1 to 10 min increased the junction density from 1.6 to 85.6 junctions/µm^2^ and the segment density from 0.8 to 29.3 segments/µm^2^. A similar trend was observed for the 30% CNT dispersion, where longer deposition times increased CNT coverage and network connectivity through the formation of additional CNT junctions and conductive segments. Because all CNT films were fabricated using the same deposition protocol, this trend is expected across all CNT dilutions.

**Figure 4:**
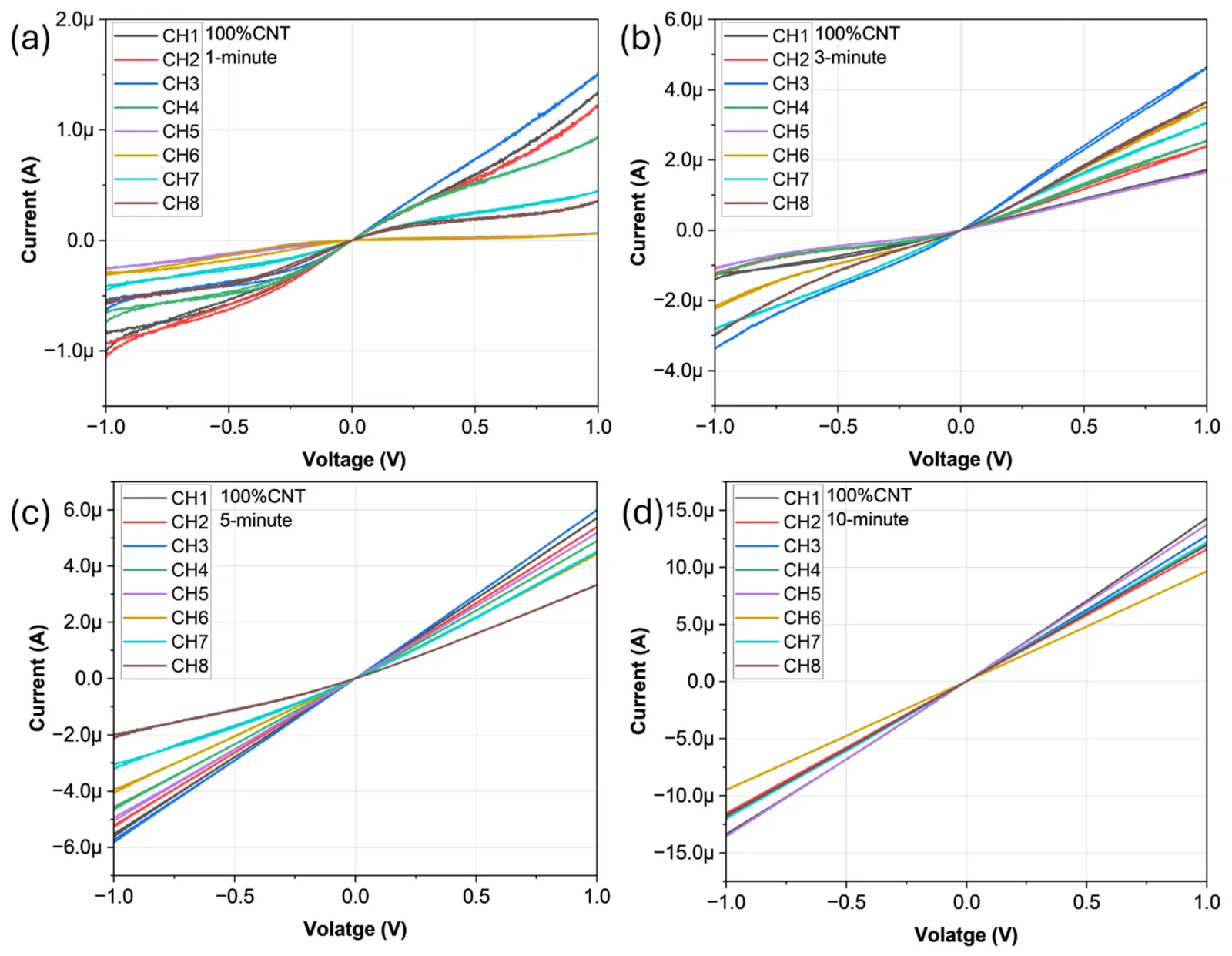
Two-terminal I-V characteristics of CNT films/channels measured without gate bias for devices fabricated with a 100% CNT concentration. (**a**) 1 min deposition time; (**b**) 3 min deposition time; (**c**) 5 min deposition time; (**d**) 10 min deposition time.

To systematically investigate the effect of CNT network density on charge transport in random CNT network films, CNT films deposited from the undiluted (100%) CNT dispersion at deposition times of 1, 3, 5, and 10 min were electrically characterized. Representative two-terminal I-V curves for CNT networks deposited for 1 min, 3 min, 5 min and 10 min at 100% CNT concentration are shown in Figure 4a–d respectively.

The 1 min CNT films exhibited the lowest current levels, ranging from approximately 250 nA to 1 µA. These films also showed non-linear I-V characteristics and noticeable variation between individual CNT films. The I-V characteristics of the 3 min CNT films, illustrated in Figure 4b, still exhibited non-linear behaviour but with higher current levels flowing through the CNT network, ranging from approximately 1 µA to 3.3 µA. Figure 4c presents the I-V characteristics of the 5 min CNT films, which displayed more linear I-V behaviour and a higher current range (~2–6 µA) compared to the 3 min films. The 10 min CNT films, being the densest among all samples, exhibited the highest current levels, spanning from approximately 10 µA to 14 µA, as shown in Figure 4d. In addition, the variation between individual channels was significantly reduced in the 10 min CNT films. Although all eight channels were fabricated using the same process, channel-to-channel variations are observed across devices with sparse CNT networks. For a low-density network, transport is dominated by a limited number of random conductive pathways. As a result, small differences in CNT arrangement between channels can lead to noticeable variations in the measured current in device with sparse CNT network. As the CNT network becomes denser with increasing deposition time, the larger number of interconnected conductive pathways reduces network variations, resulting in more consistent electrical characteristics across the channels in a device. Two-terminal I-V curves for CNT films fabricated with CNT dilutions (80%, 50%, and 30%) are shown in Figures S5–S7. Similar trends were observed across all CNT concentrations, where increasing deposition time resulted in higher current levels and more linear I-V characteristics. However, lower CNT concentrations of 50% and 30% exhibited lower current values, stronger non-linearity, and greater variation between channels, particularly at shorter deposition times. In contrast, the 80% CNT films showed improved conductivity and more consistent behaviour.

The I-V characteristics demonstrate a clear transition in charge transport behaviour with increasing CNT network density. The sparsest CNT films exhibited the lowest current levels, nonlinear I-V behaviour, and significant channel-to-channel variation, indicating that charge transport was strongly limited by CNT-CNT junctions and low-density network pathways. According to Hu et al. [34], sparse CNT networks near the percolation threshold contain only a limited number of conductive pathways, and conduction is strongly influenced by CNT-CNT junction resistance. As the CNT density increased, the current magnitude progressively increased, the I-V curves became more linear and symmetric, and the channels became more uniform, suggesting the formation of additional parallel conductive pathways throughout the network. Nirmalraj et al. reported that, in random CNT network films, a higher nanotube density creates more conductive pathways, resulting in lower junction resistance and ultimately higher overall film conductivity [35,36]. This observation aligns with the transition in charge transport behaviour observed in the I-V curves and the AFM showing an increasing number of CNT-CNT junctions as a function of deposition time in our work.

**Figure 5:**
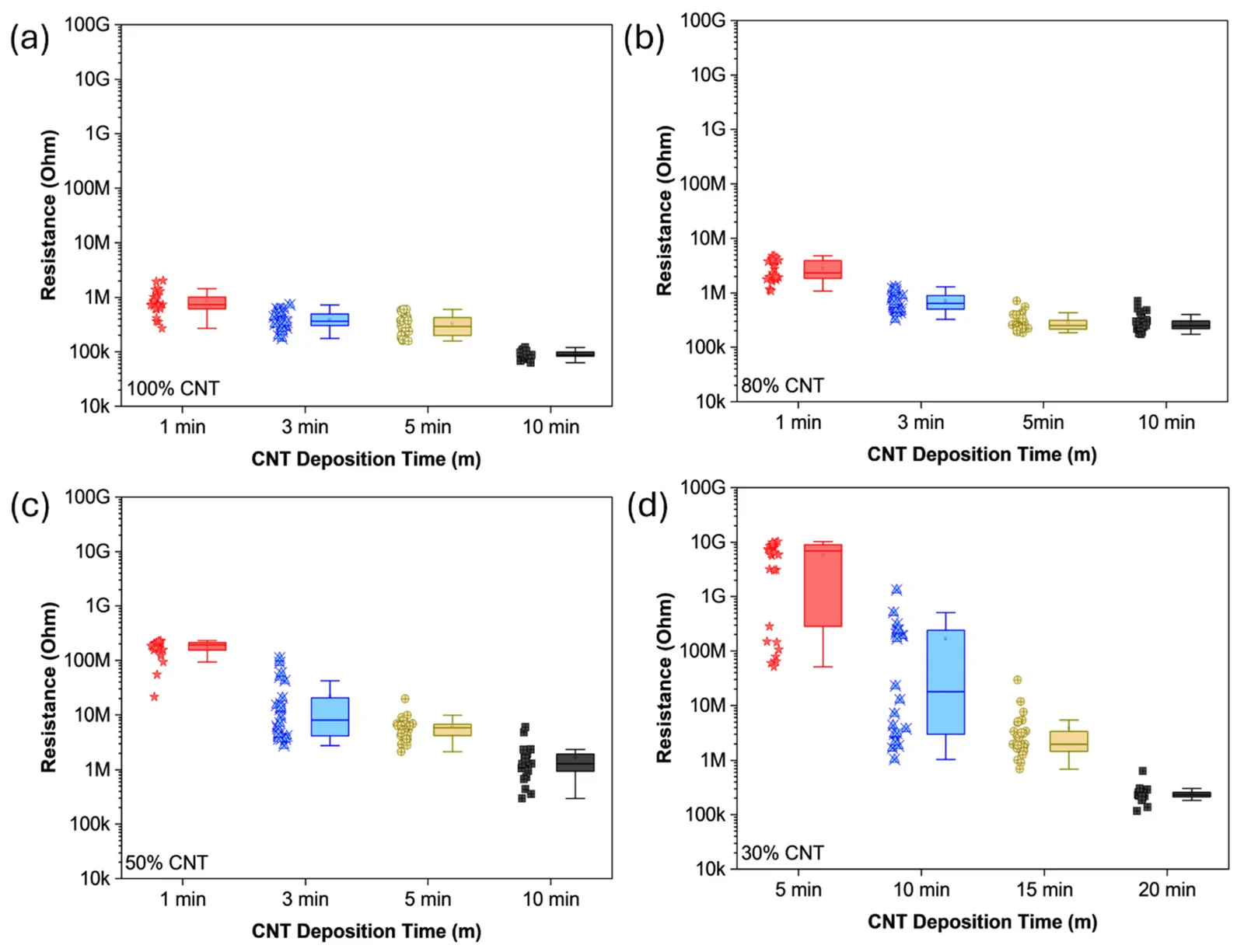
Box plots of the resistance distribution measured from CNT network films fabricated using four CNT concentrations: (**a**) 100%; (**b**) 80%; (**c**) 50%; (**d**) 30%.

To further investigate the influence of intertube junctions on the overall resistance of the random CNT network film, the resistance of the CNT films (CNT-FET channels) was extracted from the linear region of the I-V curves as described in the SI (Figure S8). Figure 5 shows the channel resistance distribution for CNT network films fabricated using undiluted 100% and the diluted 80%, 50%, and 30%, CNT dispersions at deposition times of 1, 3, 5, 10, 15, and 20 min. Each data point represents the resistance of an individual CNT-FET channel, while the box plots indicate the statistical distribution of the resistance values. The resistance of the CNT network films progressively decreased with increasing CNT deposition time in all samples, indicating improved electrical conductivity at higher CNT densities. The 100% CNT films (Figure 5a) exhibited the lowest resistance values overall, decreasing from approximately the MΩ range at 1 min to approximately 100 kΩ at 10 min. The resistance distributions also became narrower at longer deposition times, indicating reduced variability between channels. The 80% CNT films (Figure 5b) showed a similar trend, with resistance progressively decreasing as deposition time increased. Compared to the 100% CNT films, slightly higher resistance values and broader distributions were observed, particularly at shorter deposition times. The 50% CNT films (Figure 5c) exhibited substantially higher resistance. At short deposition times, resistance values extended into the multi-MΩ range, while longer deposition times reduced the resistance toward lower kΩ values. The 30% CNT films (Figure 5d) showed the highest resistance and the largest variation across all deposition times. The 1 min and 3 min films displayed very broad resistance distributions extending over several orders of magnitude, whereas the 20 min films showed a significant reduction in resistance down to kΩ with a narrower spread.

Previous experimental work has demonstrated that electrical transport in CNT networks is dominated by CNT-CNT junctions rather than the intrinsic resistance of individual nanotubes [29,30,37]. Although the AFM analysis presented here primarily characterizes the surface network, the electrical transport reflects the connectivity of the full three-dimensional CNT film, where both in-plane and through-thickness junctions contribute to the measured device performance. Fuhrer et al. demonstrated that different junction types exhibit markedly different conductance values, with metal-semiconducting (M-S) junctions introducing significantly higher resistance due to Schottky barrier formation [29]. Building on these findings, Simoneau et al. proposed that charge transport in random CNT networks is governed by the lowest-resistance percolation pathways, while high-resistance M-S junctions strongly influence overall network conductivity in near percolation random network CNT films [30]. More recently Ponzoni et al. showed that in random CNT networks, the relative macroscopic contributions of CNT intrinsic resistance and CNT-CNT junction resistance closely follow their microscopic resistance ratio, suggesting that both mechanisms should be considered when interpreting transport in CNT network devices [37]. Topinka et al. showed that both the composition and density of CNTs within a network critically determine its electrical transport properties and device performance [38].

Our results demonstrate that in the dense CNT networks obtained at longer deposition times, charge transport is primarily governed by interconnected CNT-CNT conductive pathways. Increasing deposition time increases the CNT density, promoting the formation of a larger number of interconnected CNT-CNT junctions and continuous pathways throughout the network. Consequently, the overall resistance decreases to approximately 100 kΩ as the network moves well above the percolation threshold. In contrast, the CNT network films fabricated using diluted CNT dispersion, particularly the 30% dilution CNT films, contain a much smaller number of interconnected CNT-CNT junctions. The sparse morphology limits the formation of continuous conductive pathways, resulting in transport dominated by isolated conduction pathways and contact-related effects. Under these conditions, Schottky barriers formed at the metal-CNT interfaces contribute more significantly to the overall device resistance. As a result, the measured resistance increases from 100 MΩ on a 100% CNT 1 min film to around 10 GΩ on a 30% CNT 5 min film and exhibits substantially larger channel-to-channel variation compared to the denser CNT films.

To study the impact of CNT network density on the electronic properties of CNT-FETs in an aqueous environment, CNT-FETs were encapsulated using a 1.2 µm layer of AZ1518 photoresist and electrical measurements were performed in an electrolyte-gated setup as described in Section 3.

**Figure 6:**
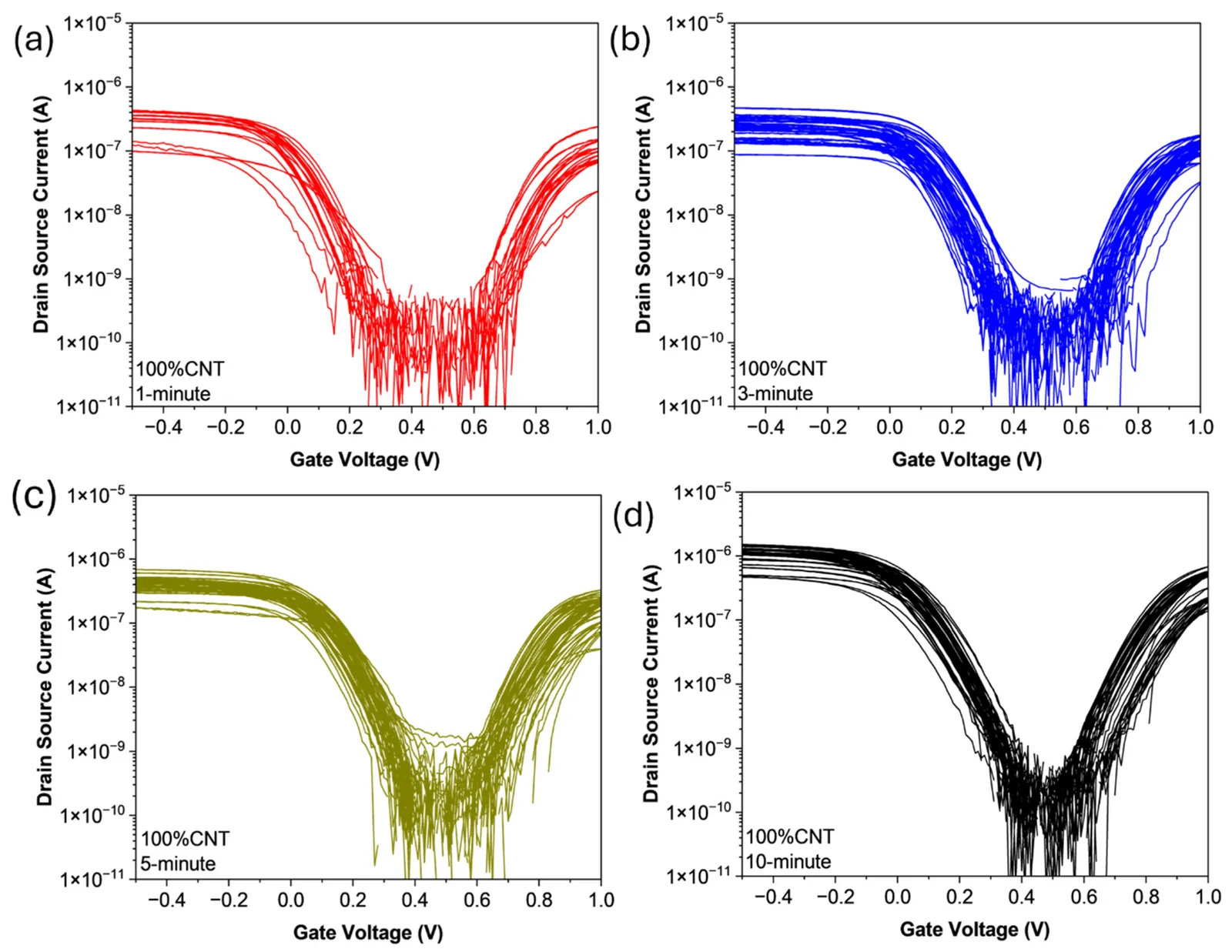
Liquid-gated transfer characteristics of CNT-FET devices fabricated using undiluted (100% CNT concentration) networks with deposition times of (**a**) 1 min, (**b**) 3 min, (**c**) 5 min, and (**d**) 10 min.

Figure 6 shows the liquid-gated transfer characteristics of CNT-FET devices fabricated using an undiluted (100%) CNT dispersion with deposition times of 1, 3, 5, and 10 min. For each deposition time, three devices were fabricated, with each device containing eight individual CNT-FETs, giving a total of 24 CNT-FETs per deposition time (96 CNT-FETs in total). Of these, 10, 21, 23, and 23 CNT-FETs were electrically functional for the 1, 3, 5, and 10 min deposition times, respectively. A CNT-FET device was classified as functional if it exhibited both a measurable drain–source current and a clear gate-dependent modulation of the channel conductance under liquid-gate operation. Typical ambipolar behaviour is observed across all devices, with conduction occurring at both positive and negative gate voltages and a minimum current region appearing near Vg ≈ 0.4–0.6 V, consistent with previous reports [39]. The fluctuations observed at low drain currents are mainly associated with measurements performed near the instrument noise floor, where small absolute current variations become visually amplified on a logarithmic scale. Similar behaviour has also been reported in CNT-FET transfer characteristics by Wang et al. [27], where comparable fluctuations are evident in the off-state region despite the devices exhibiting excellent electrical performance. CNT-FETs fabricated with 1 min CNT networks exhibit the lowest on-current, ranging from approximately 100–400 nA, whereas devices with 10 min CNT networks display the highest on-current, reaching approximately 1 µA. Increasing the deposition time from 1 to 10 min also reduces device-to-device variability, consistent with trends observed in the two-terminal I-V measurements.

Figures S9–S11 show the liquid-gated transfer characteristics (forward and reverse sweep) of CNT-FET devices fabricated using 80%, 50%, and 30% CNT concentrations at different deposition times. Similar ambipolar behaviour was observed across all devices. Increasing deposition time generally increased the on-current and reduced device-to-device variability. The fabrication yield was determined from the number of functional CNT-FETs. The fabrication yield varied with CNT concentration and deposition time, with lower CNT concentrations producing fewer functional devices and lower functional device yield likely due to the reduced CNT network. The yield of functional CNT-FETs for each CNT concentration is discussed in the SI.

**Figure 7:**
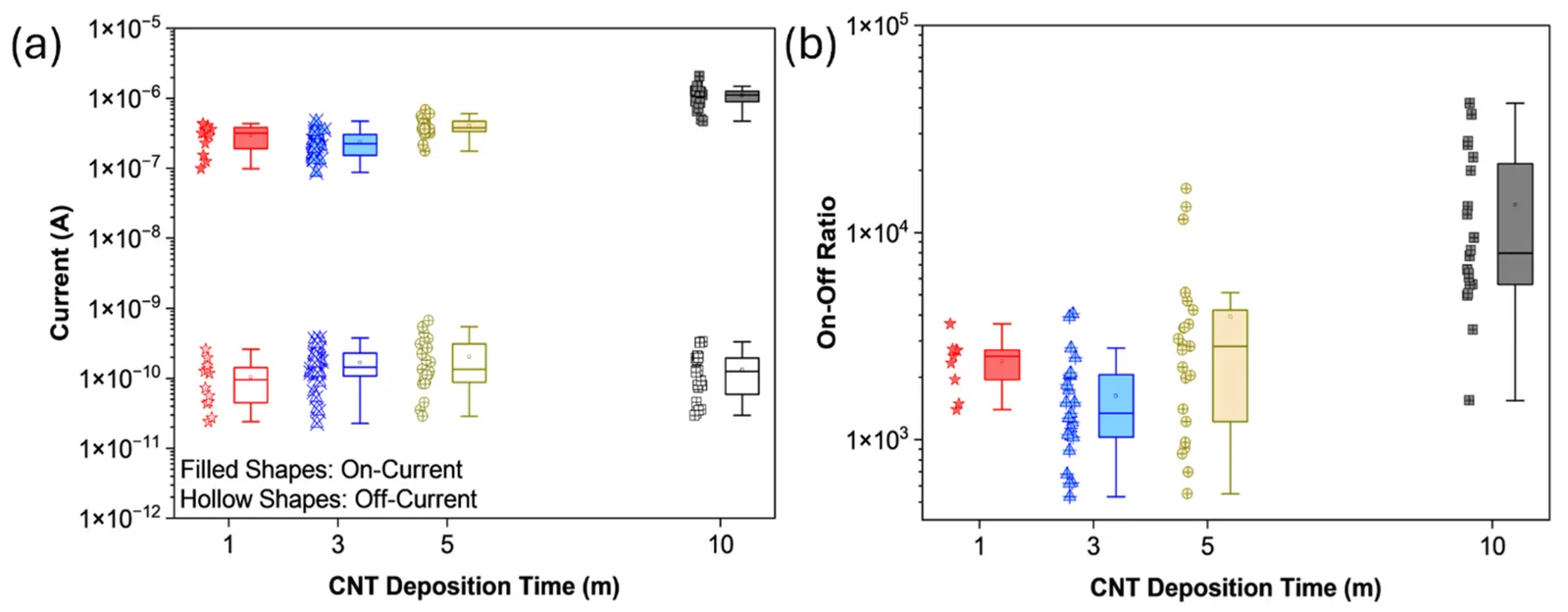
Electrical performance of CNT-FET devices fabricated using undiluted (100% CNT concentration) CNT dispersion as a function of CNT deposition time. (**a**) Distribution of on-current and off-current for devices fabricated with CNT deposition times of 1, 3, 5, and 10 min. (**b**) Corresponding on/off ratio distributions for each deposition condition.

Rosenblatt et al. reported that p-type conduction dominates CNT-FET operation under ambient conditions [8]. Under negative gate bias, hole injection is enhanced, leading to higher conductance in the p-type region. As the gate voltage becomes more positive, hole concentration decreases and conduction is suppressed as the device transitions toward n-type behaviour. However, electron transport is typically weaker because Cr/Au drain–source electrodes form p-type contacts with CNTs, creating depletion barriers and increasing contact resistance during n-type operation [8]. This behaviour is consistent with the present results, where higher current is observed at negative gate voltages and reduced conductance occurs as the gate voltage shifts toward more positive values. Gnanasekaran et al. investigated the electrical transport in CNT networks using quantitative electron tomography. They demonstrated that increasing CNT aspect ratio increases the number of CNT-CNT contacts, which leads to improved network connectivity and higher electrical conductivity [40]. Consistent with these findings, our results show a clear increase in on-current as the deposition time increases from 1 to 10 min. This trend reflects the increase in CNT network density, which provides additional conductive pathways across the channel and reduces the overall channel resistance [41,42]. This behaviour is aligned with the two-terminal I-V measurements discussed earlier, where devices fabricated with longer deposition times exhibited higher current and lower resistance. Figure 7a shows the distribution of the on- and off-currents of the CNT-FET devices fabricated using 100% CNT with different CNT deposition times. The on-current was defined as the drain current measured at a gate voltage of −0.5 V while the off-current was defined as the mean drain current over the gate voltage range of 0.4–0.6 V for each transfer characteristic. Consistent with the trends observed in the transfer characteristics, variations in the measured on-current values were observed across the different deposition conditions. The off-current was generally maintained within the range of ~10^−11^–10^−9^ A. Figure 7b presents the corresponding on/off ratio distributions, where devices fabricated with deposition times of 1–5 min displayed values predominantly within the order of 10^3^. The 10 min deposition condition resulted in an increase in the median on/off ratio with values extending toward 10^4^–10^5^. The CNT dispersion used in this work contained 95% semiconducting nanotubes and the 5% fraction of metallic CNTs cannot be avoided. Previous studies have shown that metallic nanotubes can form metallic-semiconducting junctions within randomly distributed mixed metallic and semiconducting CNT networks [38] and randomly distributed 99% semiconducting CNT network [43]. Here the 5% metallic CNTs form an integral part of the network, either as isolated CNTs or in bundles, which will impact the devices. However, the low off-currents across the devices measured in this work indicate that transport is predominantly governed by the semiconducting CNTs forming the bulk of the network. The impact of the 5% metallic fraction of the CNT network cannot be seen in other electrical characteristics such as the on-current, on/off ratios, the threshold voltage, and the subthreshold swing.

Gong et al. reported that increasing CNT network density decreases the on/off ratio because denser networks create more conductive pathways while also increasing the likelihood of metallic nanotubes participating in charge transport [41]. Our results demonstrate that the range of off-current values remains the same across deposition times and the increase observed in the on/off ratio of the 10 min film arises from the higher on-current values in dense films. Most of the on/off ratios reported in the literature are obtained from CNT-FETs operating in a back-gate configuration, where relatively high gate voltages are applied and consequently higher on/off ratios are often achieved. In contrast our results demonstrate a reasonably high on/off ratio in the range of 10^4^ while operating at significantly lower voltages, with a drain–source voltage as low as 100 mV, showing the effectiveness of the device switching under low-power operating conditions [44,45,46].

**Figure 8:**
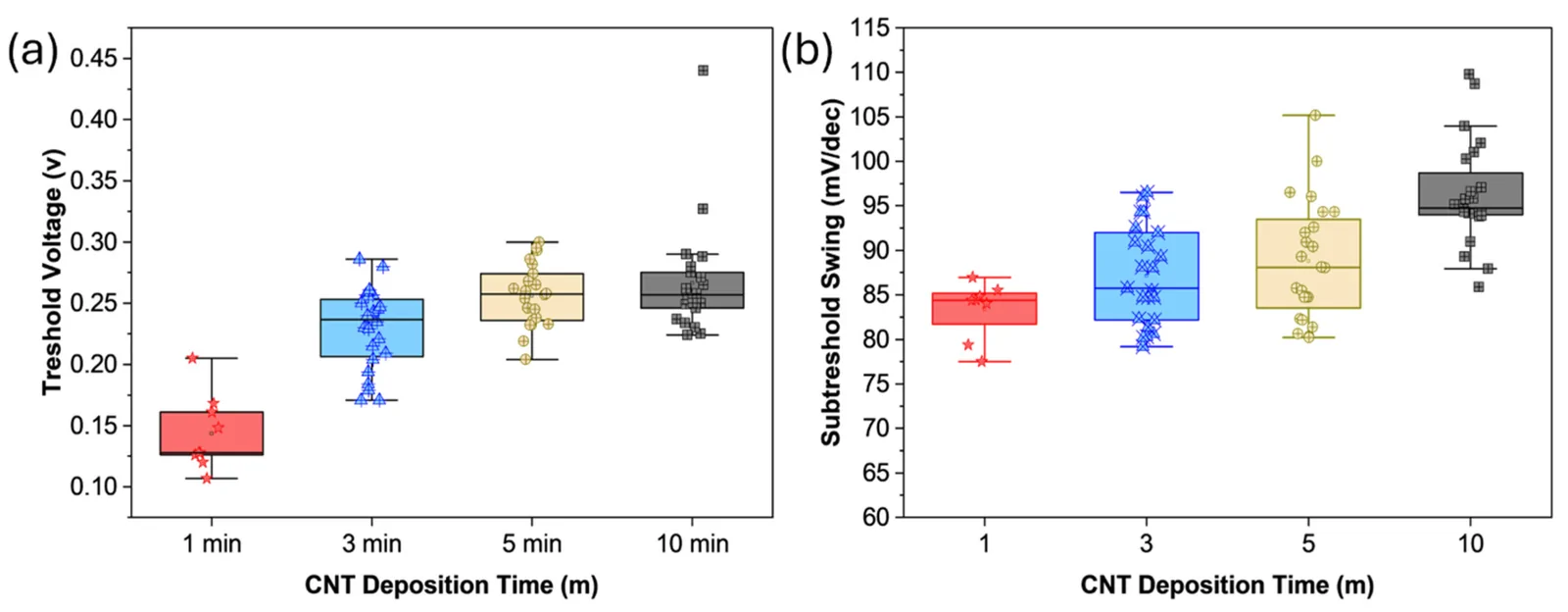
(**a**) Statistical distribution of threshold voltage (Vth) for CNT-FET devices with 100% CNT concentration as a function of CNT deposition time. (**b**) Statistical distribution of subthreshold swing (SS) for CNT-FET devices with 100% CNT concentration as a function of CNT deposition time.

In electrolyte-gated CNT-FETs, the sensing mechanism is primarily governed by electrostatic gating occurring at the CNT/electrolyte interface, where charged species present in the surrounding environment generate local electric fields that modulate the carrier concentration within the CNT channel, consequently resulting in shifts in the electrical characteristics of the device [9,10,11,31]. Therefore, to understand the influence of CNT network density on electrostatic gating behaviour, the threshold voltage (Vth) and subthreshold slope (SS) were investigated, since variations in network density can affect gate coupling efficiency and charge screening within the CNT network. Figure 8a shows the statistical distribution of the threshold voltage (Vth) as a function of deposition time for CNT-FETs with 100% CNT concentration. The threshold voltage of the CNT-FETs was determined from the transfer characteristics using the constant current extraction method [47]. Vth was defined as the gate voltage corresponding to a constant drain–source current of 15 nA. There is a visible positive correlation between deposition time and the threshold voltage. As density increases from 1 min to 10 min, the median Vth shifts from approximately 0.13 V to 0.26 V. The observed increase in Vth with CNT deposition time indicates a systematic shift in the electrostatic operating point of the device as the network becomes denser.

The threshold voltage shift suggests that a higher gate voltage is required to induce conduction, reflecting changes in the overall electrostatic potential distribution within the CNT network. In CNT-FETs with dense CNT networks, the presence of multiple interconnected nanotubes and increased capacitive interactions modifies the local electrostatic environment, effectively shifting the balance between the gate field and the intrinsic network potential. As a result, the device operates at a higher effective threshold. For sensing applications, this shifted operating point is critical since the device’s sensitivity depends on the electrical modifications of the CNT network. Therefore, in a CNT-FET with a dense CNT network, a reduced gate coupling directly suppresses the transconductance response to small charge perturbations caused by electrostatic gating mechanisms.

Figure 8b shows the variation in subthreshold swing (SS) as a function of CNT deposition time. A clear increase in SS is observed with increasing CNT density, with devices fabricated at shorter deposition times exhibiting lower SS value, indicating more efficient gate coupling. In contrast, denser CNT networks show higher SS, suggesting reduced electrostatic control over the channel. The theoretical subthreshold swing of 60 mV/dec is calculated at room temperature using

(1) $$SS=\left\lfloor \frac{k_{B}T}{e}\right\rfloor \ln10$$

where SS is the subthreshold swing and $k_{B}$, T, and e are the Boltzmann constant, the temperature, and the elemental charge, respectively [48]. Katsura et al. reported an SS of 60 mV/dec for the local electrolyte-gated individual CNT-FET which was substantially lower than the 190 mV/dec observed for the back-gated individual CNT-FET device in their study. Liu et al. reported that the use of an ionic-liquid gate CNT network FET significantly improved the SS due to the enhanced gate capacitance, reducing the SS to 75 mV/dec with an average value of ~90 mV/dec, while some devices approached the theoretical room-temperature limit of 60 mV/dec [49]. Our lowest SS value is 77.5 mV/dec with an average value of 83 mV/dec related to CNT-FETs with 100% 1 min CNT film which is lower than the SS in electrolyte-gated CNT-FETs from CNT networks in the literature [49]. Our observed increase in SS with CNT network density can be understood in terms of reduced electrostatic gate coupling arising from enhanced screening within the network. In dense CNT films, neighbouring nanotubes introduce significant electrostatic shielding and capacitive coupling, which reduce the effective gate capacitance and limit the penetration of the gate electric field to individual conduction pathways [32]. This has direct implications for electrostatic gating from charged species. In dense networks, these perturbations are screened, reducing their impact on the overall device current. Consequently, an increased SS reflects a reduced sensitivity to electrostatic changes, promoting the importance of network density in optimizing sensing mechanism [50].

CNTs contain intrinsic vacancy defects from synthesis [51] or fabrication processes that can be oxidized upon exposure to oxygen, resulting in the formation of epoxy, carbonyl, and ketene functional groups [52]. Mowbray et al. [52] showed that these oxygen-containing defect sites significantly influence the electronic properties of SWCNTs. In addition to the oxygen introduced through deliberate oxidation, Wepansik et al. [53] suggested that surface oxidation can also occur unintentionally after CNTs are released into the environment through exposure to natural oxidizing agents. Furthermore, the surface oxygen content of pristine CNTs may increase through photooxidation with UV exposure, which could originate from the microfabrication processes used in the device processing, promoting the formation of oxygen-containing functional groups [54]. The presence of oxygen-containing functional groups on CNT surfaces is particularly important for sensing applications because these groups can interact with ionic species in the surrounding environment [55,56]. However, we note that all the “as-bought” CNTs here have been similarly treated and are from the same original batch with low defects, so it is assumed that the surface chemistry is nominally the same on average for all of the CNTs.

Here we investigate the role of the CNT network morphology in determining the pH sensing performance of CNT-FETs. The pH response of CNT-FETs is primarily attributed to changes in the charge state of ionizable surface groups located at or near the CNT/electrolyte interface [15,16]. Back and Shim [15] showed that decreasing the solution pH causes a negative shift in the threshold voltage while the transconductance remains unchanged, indicating that lower pH decreases the hole concentration in the nanotube rather than causing proton-induced hole doping. Measurements using suspended nanotubes also ruled out the SiO_2_ substrate, suggesting that chargeable species on the nanotube surface are responsible for the pH response. Heller et al. [16] proposed that negatively charged ionizable groups on the carbon surface and underlying substrate electrostatically gate the nanotube. At low pH, protonation neutralizes these negative charges, reducing the surface potential and shifting the transfer characteristics toward more negative gate voltages. While the previous studies focus on the pH sensing mechanism in a single CNT-FET, a similar mechanism was reported by Haeberle et al. [17] for semiconducting CNT network transistors. Increasing pH promotes deprotonation, leaving more negatively charged surface sites that electrostatically modulate the CNT channel, whereas decreasing pH increases protonation, neutralizes these charges, and shifts the transfer characteristics in the opposite direction.

**Figure 9:**
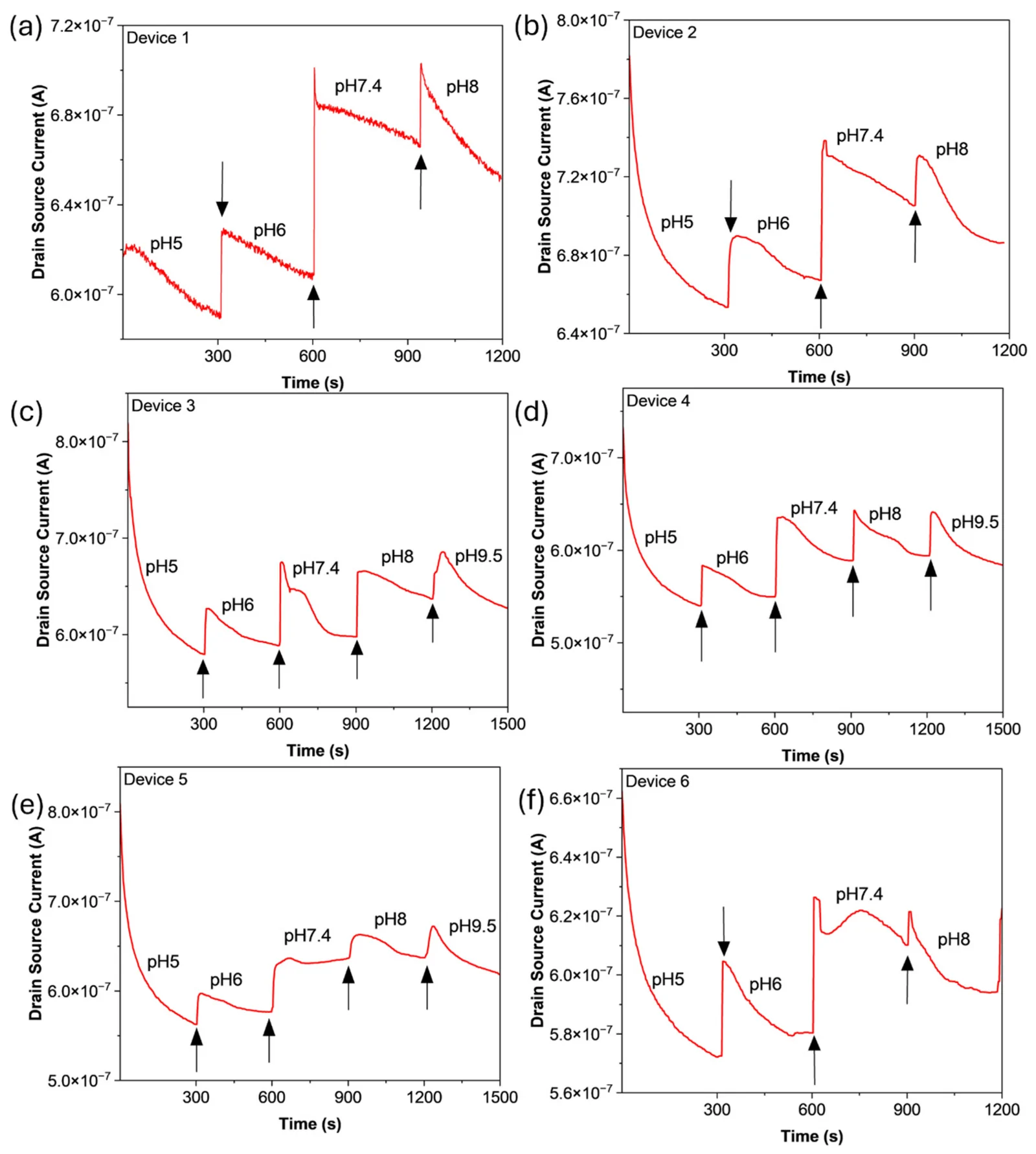
Real-time drain–source current (Ids) response of highly dense CNT-FETs with 100% 10 min CNT films for all devices upon sequential exposure to buffer solutions with increasing pH (5, 6, 7.4, 8, and 9.5) under constant bias conditions. The upward and downward arrows indicate the points at which the pH of the solution was changed during the sensing measurements. (**a**) Device 1 response. (**b**) Device 2 response. (**c**) Device 3 response. (**d**) Device 4 response. (**e**) Device 5 response. (**f**) Device 6 response. For clarity, some devices were measured only up to pH 8 because the pH 9.5 step was introduced later in the study.

**Figure 10:**
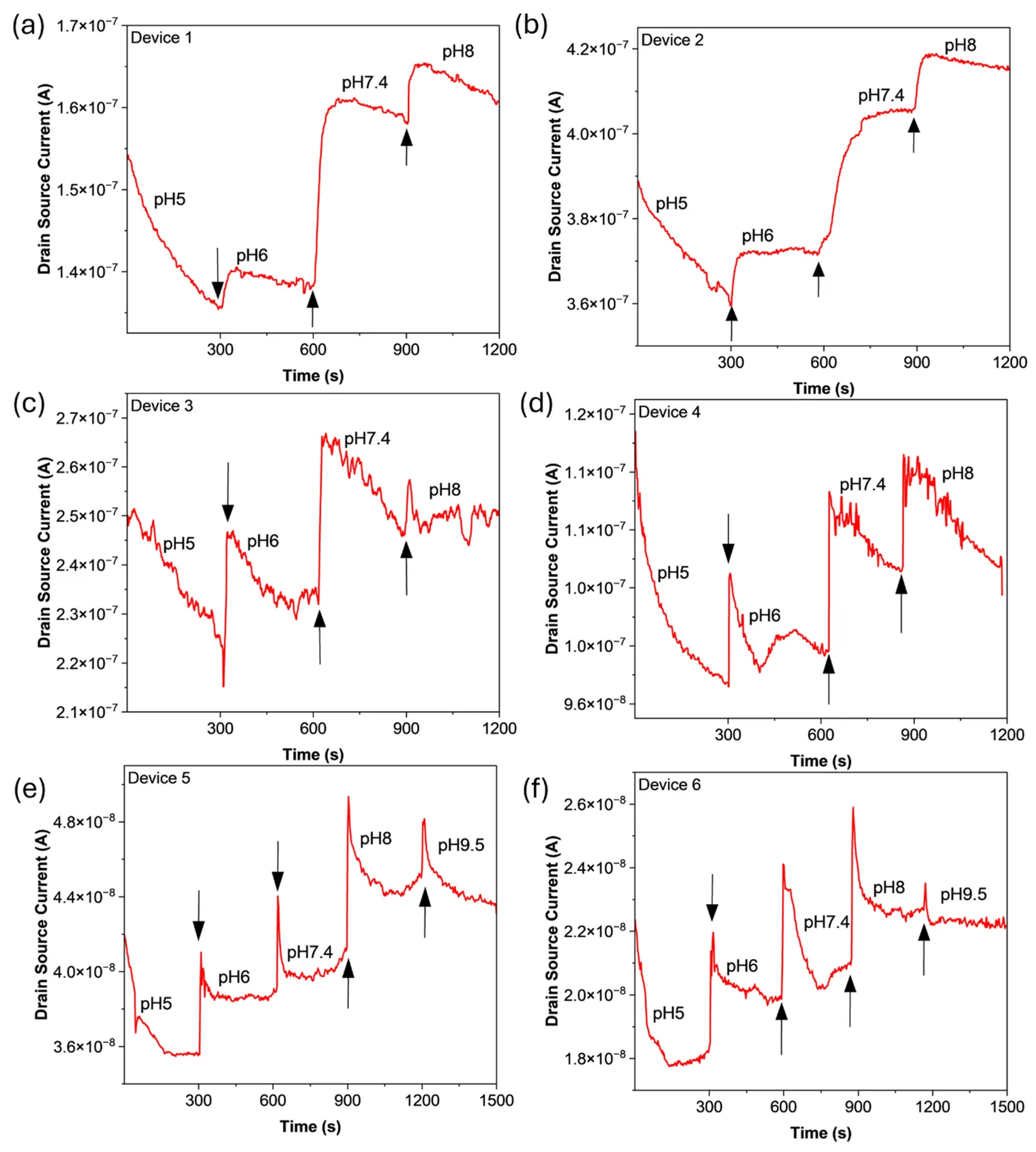
Real-time drain–source current (Ids) response of medium-density CNT-FETs with 50% 5 min CNT film for devices 1, 2, and 3 and 100% 3 min CNT film for devices 4, 5, and 6 upon sequential exposure to buffer solutions with increasing pH (5, 6, 7.4, 8 and 9.5) under constant bias conditions. The upward and downward arrows indicate the points at which the pH of the solution was changed during the sensing measurements. (**a**) Device 1 response. (**b**) Device 2 response. (**c**) Device 3 response. (**d**) Device 4 response. (**e**) Device 5 response. (**f**) Device 6 response. For clarity, some devices were measured only up to pH 8 because the pH 9.5 step was introduced later in the study.

**Figure 11:**
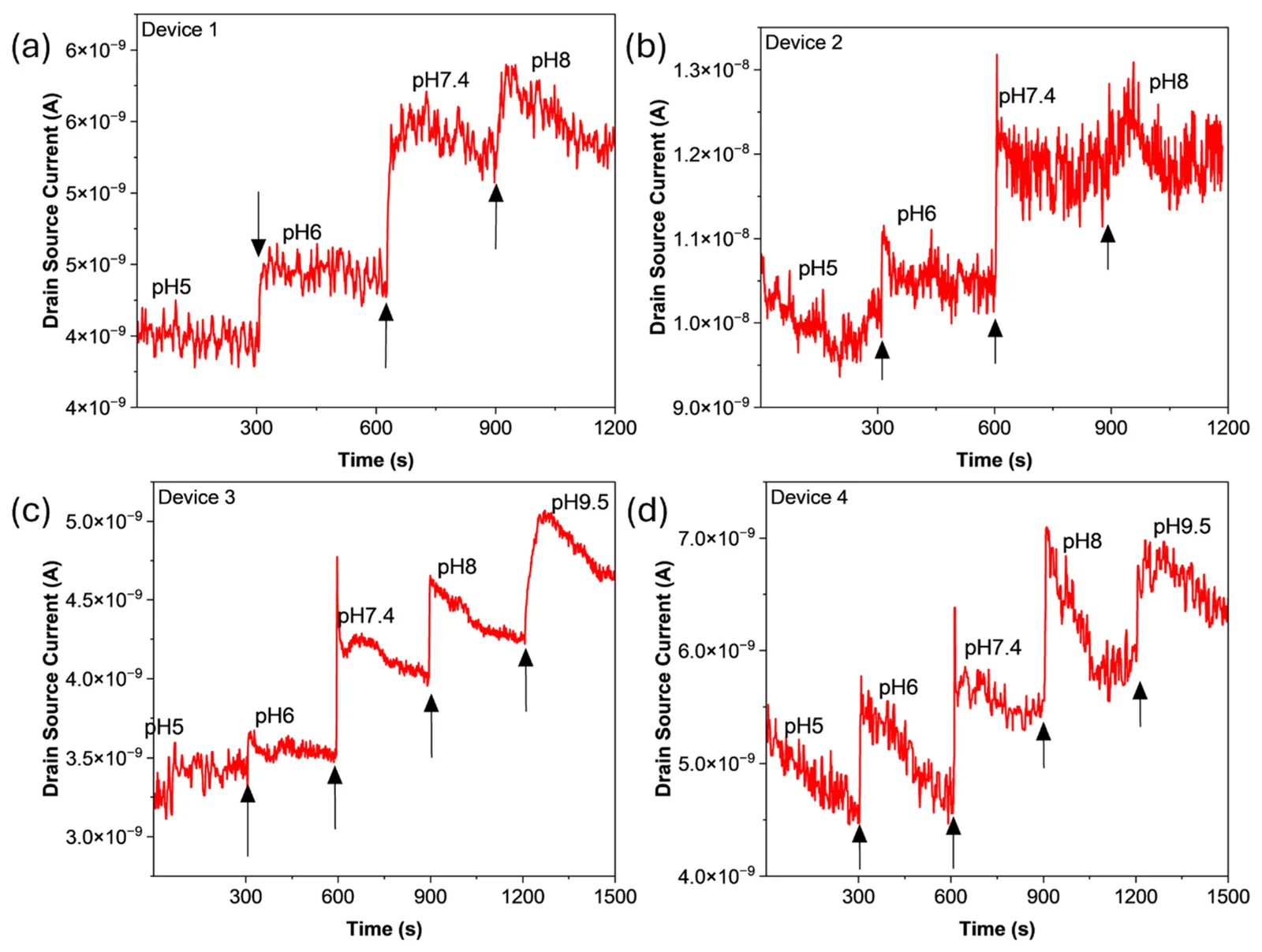
Real-time drain–source current (Ids) response of low-density CNT-FETs with 50% 3 min CNT film for device 1 and 30% 10 min CNT film for devices 2, 3, and 4 upon sequential exposure to buffer solutions with increasing pH (5, 6, 7.4, 8, and 9.5) under constant bias conditions. The upward and downward arrows indicate the points at which the pH of the solution was changed during the sensing measurements. (**a**) Device 1 response. (**b**) Device 2 response. (**c**) Device 3 response. (**d**) Device 4 response. For clarity, some devices were measured only up to pH 8 because the pH 9.5 step was introduced later in the study.

pH measurements through a series of CNT-FET devices spanning low-density, medium-density, and highly dense network regimes were investigated under varying pH conditions. Recent electrolyte-gated CNT-FET pH sensors commonly employ PBS as the electrolyte [24,26,57]. PBS provides an ionic environment for stable transistor operation [58,59]. PBS is also nontoxic and regulates changes in pH and stabilizes the biomolecules and proteins for biosensor applications [60,61]. Each device was exposed to 1X PBS solutions with pH values of 5, 6, 7.4, 8, and 9.5, while the corresponding changes in drain–source current were recorded under a fixed CNT-FET bias configuration. The pH sensing results are presented in Figure 9, Figure 10 and Figure 11 for highly dense, medium-density, and low-density network CNT films respectively. The low-density CNT network films correspond to 50% 3 min CNT and 30% 10 min CNT films. The medium-density networks correspond to 50% 5 min and 100% 3 min CNT films, and the highly dense networks correspond to a 100% 10 min CNT film. The drain–source current (Ids) across all devices clearly demonstrates successful pH sensing with different CNT network densities.

The increase in drain–source current observed in CNT-FETs as the pH of the electrolyte is raised from 5 to 9.5 can be attributed to the modulation of surface charges at the CNT/electrolyte interface. As pH increases, the decrease in proton concentration promotes the deprotonation of ionizable surface groups at or near the CNT surface resulting in an increase in negative surface charge. The increased surface charge modifies the surface potential, producing an electrostatic gating effect that enhances hole conduction in the p-type CNT-FET, thereby increasing Ids current [24,27,62].

Devices with highly dense CNT networks displayed in Figure 9 exhibit higher absolute drain current responses upon pH exposure. They also suffer from pronounced downward baseline drift. Similar baseline drift during real-time pH measurements has also been observed in previous CNT-based pH sensing studies, indicating that this behaviour is not unique to the present devices [24,63]. We attribute the more pronounced accumulation observed in the dense CNT network devices to an electrostatic screening effect within the highly interconnected film, as discussed previously. In contrast, this behaviour is substantially reduced for the medium-density and low-density network devices, where electrolyte gating is more effective and the current response more closely follows the applied pH changes. Despite this instability, the noise level in highly dense CNT-FETs remains extremely low as the absolute current value ranges between ~500 to ~800 nA, which can be attributed to the large number of interconnected conduction pathways and improved charge transport within the dense CNT network.

Compared with highly dense CNT networks, devices with medium-density CNT networks, as shown in Figure 10, exhibit a more balanced sensing behaviour, with clearer stepwise responses to pH changes. In addition, the devices still preserve relatively low noise levels due to the presence of sufficient interconnected conduction pathways, while avoiding the excessive electrostatic screening observed in highly dense networks. The absolute current range in these devices is lower than the highly dense devices ranging between ~20 to ~400 nA.

Devices with low-density CNT networks displayed in Figure 11 exhibit the lowest absolute current levels of under 10 nA and the highest noise among all network densities due to the limited number of conductive pathways and the dominance of individual CNT-CNT junctions in charge transport. The reduced connectivity within sparse networks makes electrical conduction highly sensitive to local fluctuations, resulting in increased signal noise.

**Figure 12:**
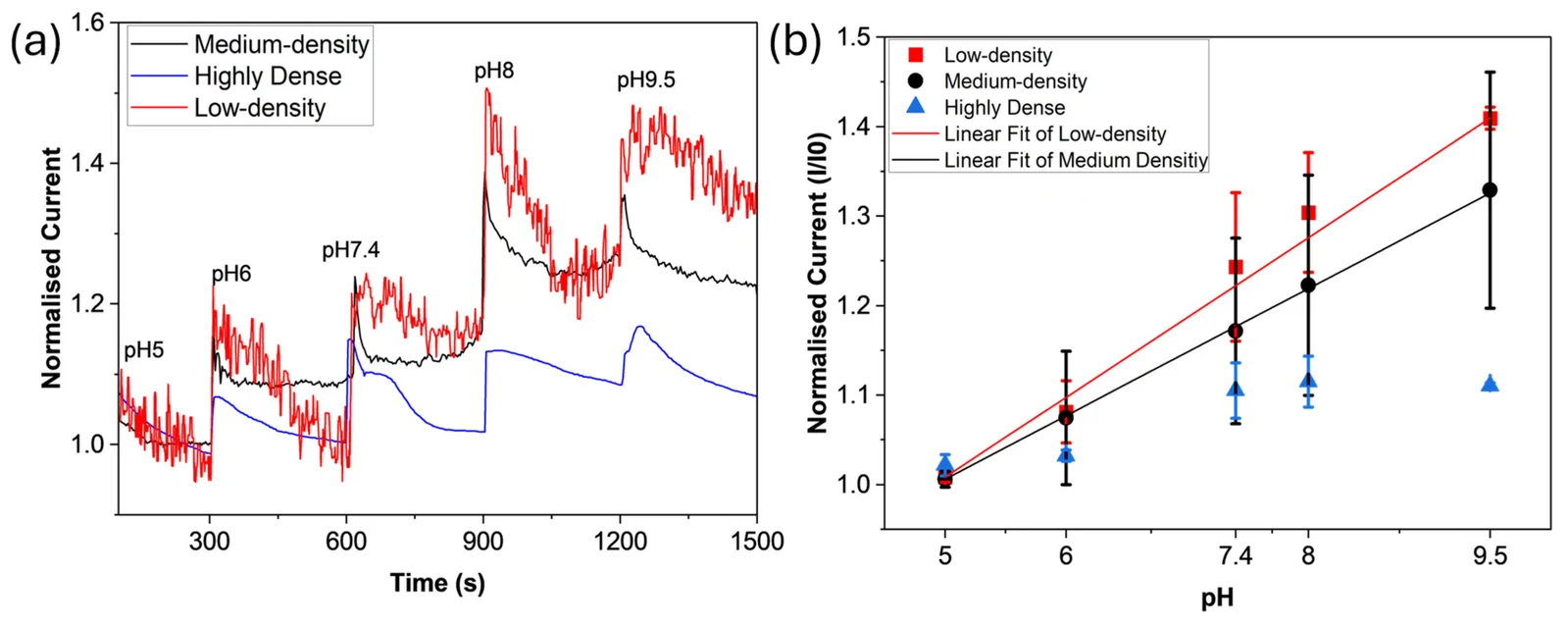
(**a**) Real-time normalized current response of CNT-FET devices with different network densities (low density: 30% 10 min CNT film, medium density: 100% 3 min CNT film, and highly dense: 100% 10 min CNT film) to stepwise changes in pH. (**b**) Normalized conductance response of CNT-FETs with different CNT network densities as a function of pH.

To enable a comparison of sensing responses independent of baseline current variations, the real-time pH sensing data were normalized for all devices. Representative normalized real-time responses for low-density, medium-density, and highly dense CNT networks are shown in Figure 12a. The normalized real-time sensing response further highlights the strong dependence of electrostatic modulation on CNT network density. The low-density device exhibits the largest relative conductance change upon pH variation to each buffer exchange. In contrast, the highly dense network shows a significantly reduced modulation, despite maintaining a stable and noise-free signal. This behaviour arises from the enhanced sensitivity of low-density networks to local electrostatic perturbations, as charge transport is dominated by a small number of critical conduction pathways, less screening effects and better gate coupling are confirmed via SS and Vth analysis. In contrast, highly dense networks contain numerous parallel transport channels and have enhanced charge screening within the CNT network, which effectively screen electrostatic effects and reduce the overall relative conductance modulation. Devices with medium CNT density display intermediate characteristics, reflecting a transition from low-density transport to more dense network conduction behaviour.

Figure 12b shows the normalized current response of CNT-FET devices with different CNT network densities as a function of pH. All devices exhibited an increase in normalized current with increasing pH; however, the response strongly depended on CNT network density. Low-density network devices showed the highest sensitivity, with a slope of 0.0892, corresponding to 8.9%/pH. Medium-density networks exhibited a lower sensitivity of 7.1%/pH, while highly dense networks showed the smallest response of 2.1%/pH. The reduction in sensitivity with increasing CNT density suggests that sparse CNT networks are more strongly influenced by electrostatic gating and interfacial charge modulation, whereas highly dense networks suppress the sensing response aligned with the earlier CNT-FET electrostatic gating study results. The linearity of the pH response was evaluated by linear regression analysis of the normalized current as a function of pH. The low-density network CNT-FETs exhibited the highest degree of linearity, with an R^2^ value of 0.999, indicating an excellent linear relationship between the normalized current and pH. The medium-density devices also displayed a strong linear response, although with a lower sensitivity than the low-density network devices. In contrast, the highly dense CNT networks exhibited a non-linear response, with the normalized current tending toward saturation at higher pH values. Consequently, a linear fit was not applied to these high-density devices, as their behaviour is not adequately described by a simple linear model across the investigated pH range.

Only a very small number of previous studies have addressed the limitation of long-term pH sensing stability associated with CNT-FET pH sensors, and it is under device architecture with encapsulation. Specifically. Yan et al. [64] evaluated the long-term stability of their HfO_2_-passivated CNT-FET ion-sensing platform, including the pH sensor, over 30 days. The sensitivity of the pH sensor remained nearly 70 mV/dec over the period. In another study where the focus was on device stability rather than the pH sensitivity, Jiang et al. [62] demonstrated long-term device stability by measuring the transfer characteristics, off-state current, and threshold voltage over 16 days, with negligible performance degradation. Repeatability and cyclic experiments are often included in CNT-FET pH studies [27,62,64] where the authors demonstrate repeatability and cyclic performance of usually 1 device reporting negligible changes. However, we have not conducted any long-term stability or cycling experiments but have shown that the pH sensing performance was assessed using multiple independently fabricated devices within each CNT network density category. The devices showed consistent sensitivity and similar trends in response to pH variations which implies the reproducibility of the devices in this study.

**Figure 13:**
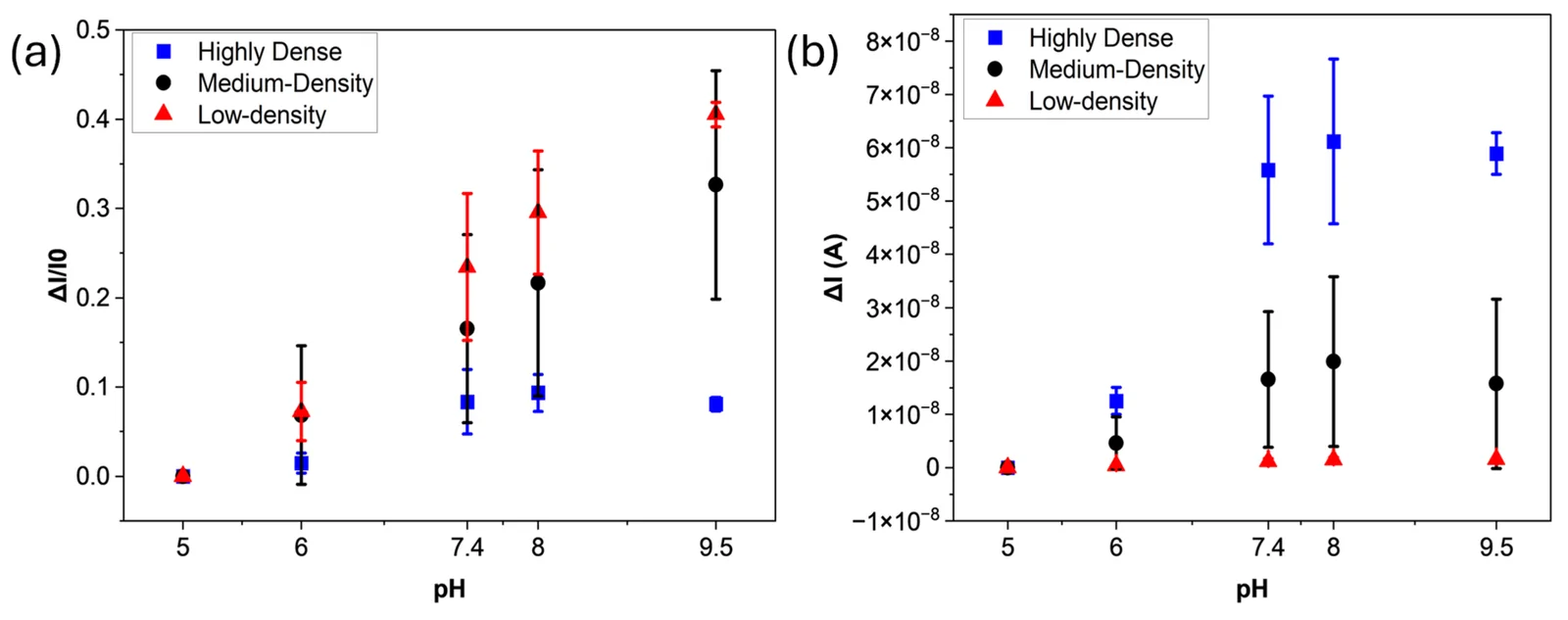
Comparison of (**a**) absolute current change (ΔI) and (**b**) normalized response (ΔI/I_0_) as a function of pH for CNT-FET devices with low-density, medium-density, and highly dense CNT networks.

For the sensing experiments, six highly dense CNT network devices, six medium-density CNT network devices, and four low-density CNT network devices were tested. The extracted absolute current change (ΔI) and normalized response (ΔI/I_0_) were subsequently analyzed to quantitatively compare the sensing performance across CNT network densities, as shown in Figure 13. Delta I values are presented as the mean, with error bars indicating the minimum and maximum values at each pH. The absolute current change exhibits a clear increase with CNT network density, with highly dense devices showing the largest signal magnitude across all pH values reaching nearly 80 nA, followed by medium-density and low-density networks.

This behaviour is expected, as dense networks provide a higher baseline conductance and a greater number of parallel conduction pathways, resulting in a larger absolute modulation of current. In contrast, the normalized response (ΔI/I_0_) follows the opposite trend, with low-density network devices exhibiting the highest relative conductance change reaching 0.4 which is a 40% increase in signal, while highly dense networks show the lowest response. The medium-density CNT network offers a balanced performance for pH sensing applications. It generates sufficiently large absolute current to reduce noise and improve signal stability, while preserving a higher sensitivity than highly dense networks, which are limited by electrostatic screening effects. These findings further confirm the impact of charge screening and gate coupling across CNT networks, while highlighting a fundamental trade-off between sensitivity and signal robustness in CNT-based pH sensors. The observed dependence of pH sensitivity on CNT network density suggests that electrostatic gating is the dominant sensing mechanism in the present devices. While pH-dependent surface chemistry may modify the charge state of surface groups, these changes are expected to influence the device primarily through electrostatic modulation of the CNT channel. Although a contribution from direct charge transfer cannot be completely excluded, the present results do not provide evidence that it is the dominant mechanism.

## 5. Conclusions

In conclusion, CNT network density strongly governs the electrical and sensing behaviour of electrolyte-gated CNT-FETs. Increasing CNT density improved conductivity, reducing channel resistance from the GΩ range to ~100 kΩ and increasing on-current up to ~1 µA, while devices maintained low operating voltages and achieved on/off ratios up to 10^4^–10^5^ with subthreshold swings as low as 77.5 mV/dec. However, higher CNT densities weakened electrostatic gate coupling through charge screening. Low-density network devices exhibited the highest pH sensitivity (8.9%/pH) and largest normalized response (~40%), whereas highly dense networks showed the lowest sensitivity (2.1%/pH) despite generating the largest absolute current change of ~70 nA. Medium-density networks provided the best balance between sensitivity, noise, and signal stability. These results demonstrate that CNT networks have a significant impact on electrostatic gating and consequently pH sensing performance. With an optimized CNT network, future work will focus on surface characterization of the CNT functional groups, as well as direct functionalization strategies that further improve the sensitivity and the detection range. Approaches to enhance long-term durability and stability of the sensors, such as encapsulation should also be explored to work towards real-world practical applications.

## Data Availability

The original contributions presented in this study are included in the article/Supplementary Materials. Further inquiries can be directed to the corresponding author.

## Supplementary Materials

The following supporting information can be downloaded at: https://www.mdpi.com/article/10.3390/s26165100/s1, Figure S1: AFM images of the deposited CNT network films of 50% CNT concentration after (a) 1 min and (b) 3 min deposition time. Corresponding binary AFM image overlaid with the analysed CNT network results are shown for 1 min CNT network film in (c) and 3 min CNT network film in (d). Figure S2: AFM images of the deposited CNT network films of 50% CNT concentration after (a) 5 min and (b) 10 min deposition time. Corresponding binary AFM image overlaid with the analysed CNT network results are shown for 5 min CNT network film in (c) and 10 min CNT network film in (d). Figure S3: AFM images of the deposited CNT network films of 30% CNT concentration after (a) 5 min and (b) 7 min deposition time. Corresponding binary AFM image overlaid with the analysed CNT network results are shown for 5 min CNT network film in (c) and 7 min CNT network film in (d). Figure S4: AFM images of the deposited CNT network films of 30% CNT concentration after (a) 10 min and (b) 15 min deposition time. Corresponding binary AFM image overlaid with the analysed CNT network results are shown for 10 min CNT network film in (c) and 15 min CNT network film in (d). Figure S5: Two-terminal I-V characteristics of CNT Films/Channels measured for devices fabricated with 80% CNT. (a) 1-min deposition time. (b) 3-min deposition time. (c) 5-min deposition time. (d) 10-min deposition time. Figure S6: Two-terminal I-V characteristics of CNT Films/Channels measured for devices fabricated with a 50% CNT. (a) 1-min deposition time. (b) 3-min deposition time. (c) 5-min deposition time. (d) 10-min deposition time. Figure S7: Two-terminal I-V characteristics of CNT Films/Channels measured for devices fabricated with a 30% CNT. (a) 5-min deposition time. (b) 10-min deposition time. (c) 15-min deposition time. (d) 20-min deposition time. Figure S8: (a) Full I-V characteristics of the 1-min CNT network film measured from −1 to 1 V, showing non-linear charge transport behaviour. (b) Linear fit applied to the low-bias region of the I-V sweep, demonstrating approximately linear behaviour with a slope of 1.38967 × 10^−6^ and an R^2^ value of 0.99983. Figure S9: Liquid-gated transfer characteristics of CNT-FET devices fabricated using 80% CNT concentration at deposition times of 1, 3, 5, and 10 min. Figure S10: Liquid-gated transfer characteristics of CNT-FET devices fabricated using 50% CNT concentration at deposition times of 3, 5, and 10 min. Figure S11: Liquid-gated transfer characteristics of CNT-FET devices fabricated using 30% CNT concentration at deposition times of 10, 15, and 20 min.
