# Supplementary material for: CNT Network Impacts on Electrolyte-Gated Carbon Nanotube Field-Effect Transistors pH Sensors
Source: Sensors (Basel). 2026 Aug 12;26(16):5100. doi: 10.3390/s26165100 (PMC13517855; doi:10.3390/s26165100)
Supplement: Supplementary file 1 [file sensors-26-05100-s001.zip › sensors-4438722-supplementary.pdf]

Supporting Information (SI)

CNT Network Impacts on Electrolyte-Gated Carbon Nanotube Field-Effect Transistors pH Sensors

Alireza Zare[1,2], Danica Fontein[1,2], Colm Carraher[3], Natalie O. V. Plank[*,1,2]

Affiliations

[1] School of Chemical and Physical Sciences, Victoria University of Wellington, Wellington 6021, New Zealand.

[2] The MacDiarmid Institute for Advanced Materials and Nanotechnology, Wellington 6021, New Zealand.

[3] Scentian Bio Ltd., Auckland 1025, New Zealand.

* Correspondence author: natalie.plank@vuw.ac.nz

1. CNT Network Junction and Segment Density Analysis

Junction and Segment density analysis was performed on the Atomic Force Microscopy (AFM) images as described by Begzaad et al. [1] Representative AFM images for carbon nanotube (CNT) films deposited for 1 and 3 min with 50% CNT concentration are shown in **Figure S1a** and **b**. Corresponding CNT network film analysis results are shown in **Figure S1c** and **d**, respectively. For 1 min CNT network film, only a limited number of CNTs are observed and the CNTs are isolated and sparsely distributed resulting in a very few CNT-CNT junctions. Consequently, the processed image shows only $1.6\ \mathrm{Junctions}/\mu\mathrm{m}^2$ and $0.8\ \mathrm{Segments}/\mu\mathrm{m}^2$ indicating the absence of a continues CNT network. For the 3 min CNT network, the surface coverage has slightly increased. The greater number of CNTs leads to more frequent crossings and contacts among neighbouring CNTs. This is reflected in the processed image by the increased number of junctions and segments to $15.7\ \mathrm{Junctions}/\mu\mathrm{m}^2$ and $7.4\ \mathrm{Segments}/\mu\mathrm{m}^2$ accordingly.

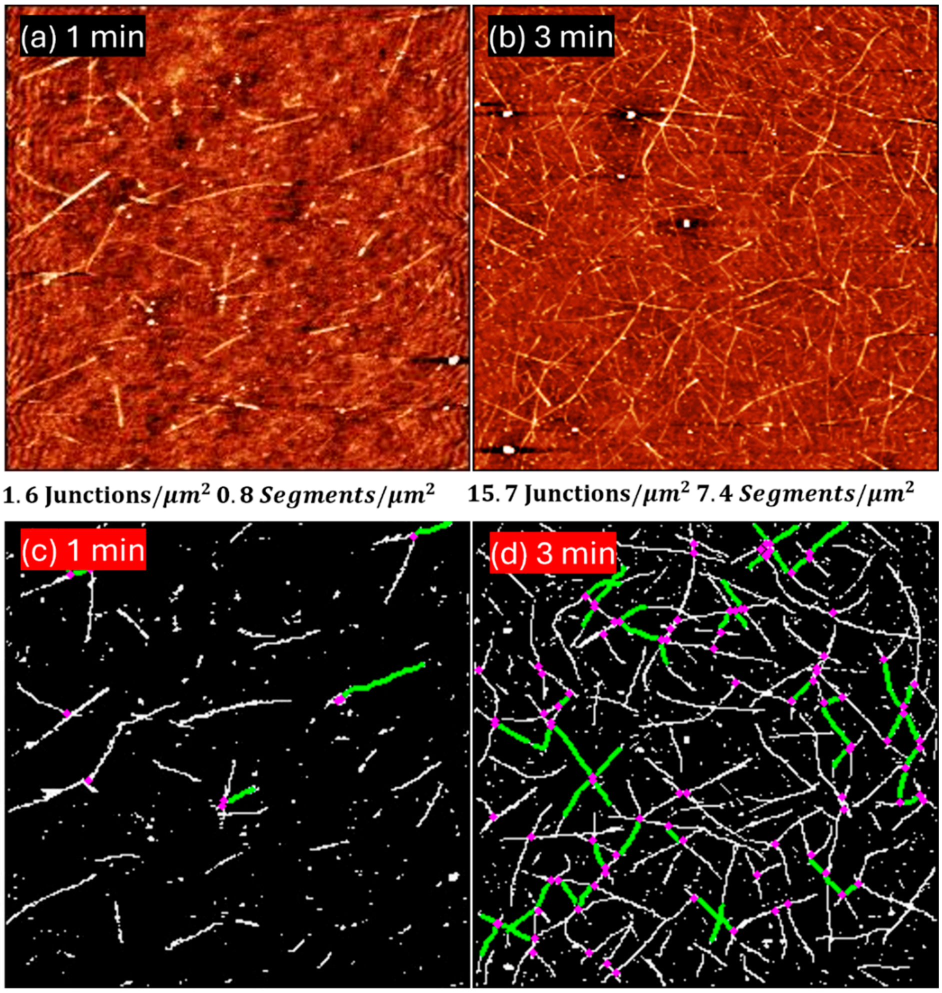

Figure S1: AFM images of the deposited CNT network films of 50% CNT concentration after (a) 1 min and (b) 3 min deposition time. Corresponding binary AFM image overlaid with the analysed CNT network results are shown for 1 min CNT network film in (c) and 3 min CNT network film in (d).

Representative AFM images for CNT films deposited for 5 and 10 min with 50% CNT concentration are shown in **Figure S2a** and **b,** while Corresponding CNT network film analysis results are shown in **Figure S2c** and **d**. At 5 min CNT deposition time deposition time, the CNT density increases further, producing a more interconnected network. Numerous CNT-CNT junctions are visible, and the network analysis reveals a significant rise in both junction and segment density of $29.8\ \mathrm{Junctions}/\mu\mathrm{m}^2$ and $10.1\ \mathrm{Segments}/\mu\mathrm{m}^2$ respectively. At 10 min CNT deposition time, the network becomes densely populated with CNTs, forming a continuous network. The number of junctions reaches its highest level at $85.6\ \mathrm{Junctions}/\mu\mathrm{m}^2$ and extensive interconnected segments of $29.3\ \mathrm{Segments}/\mu\mathrm{m}^2$ span the entire analysed area.

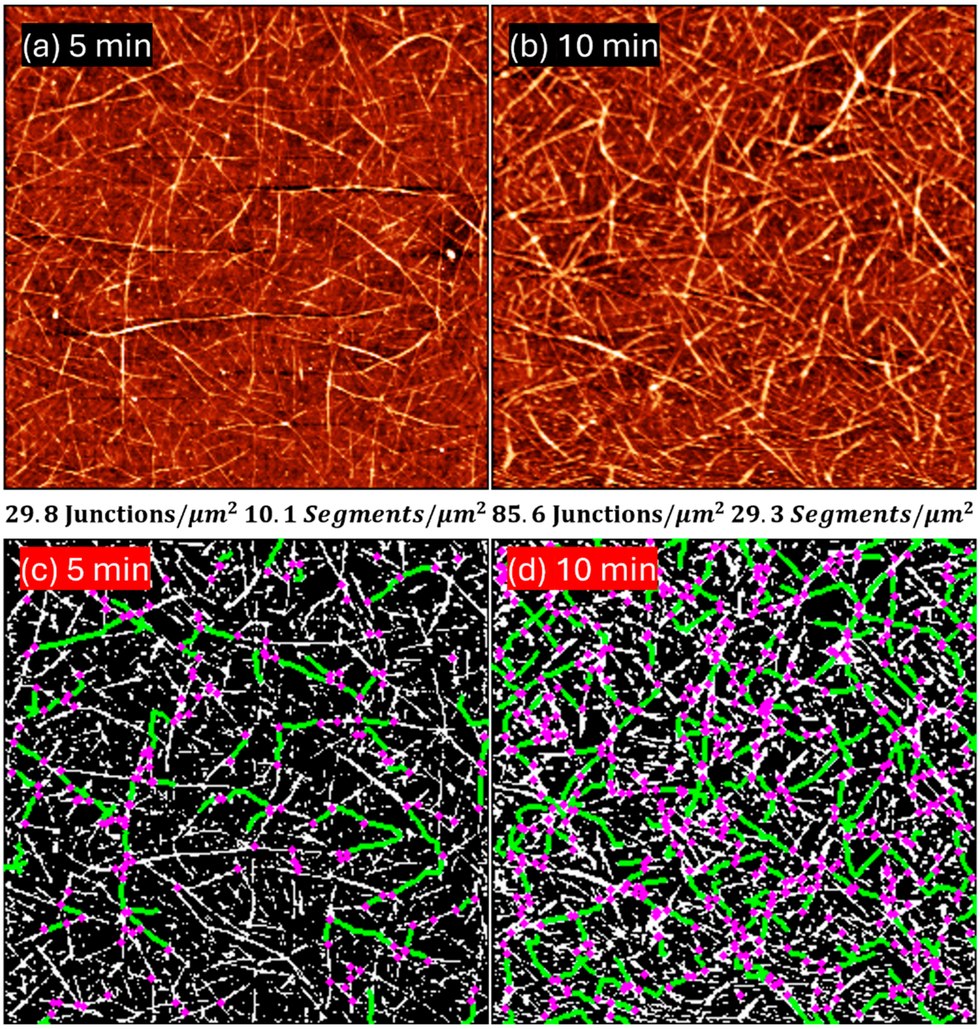

Figure S2: AFM images of the deposited CNT network films of 50% CNT concentration after (a) 5 min and (b) 10 min deposition time. Corresponding binary AFM image overlaid with the analysed CNT network results are shown for 5 min CNT network film in (c) and 10 min CNT network film in (d).

Representative AFM images of CNT films deposited from a 30% CNT dispersion for 5 and 7 min are presented in **Figure S3a** and **b**, while the corresponding CNT network analysis results are shown in **Figure S3c** and **d**. At a CNT deposition time of 5 min, the 30% CNT film exhibits a sparse distribution of nanotubes, with only a small number of junctions calculated as $1.1\ \mathrm{Junctions/\mu m^2}$ observed throughout the analysed area. Most CNTs remain isolated. Increasing the deposition time to 7 min results in a denser network, with more frequent nanotube overlap and a corresponding an increase of $4.3\ \mathrm{Junctions/\mu m^2}$ in junction and $2.2\ \mathrm{Segments/\mu m^2}$ in segment density.

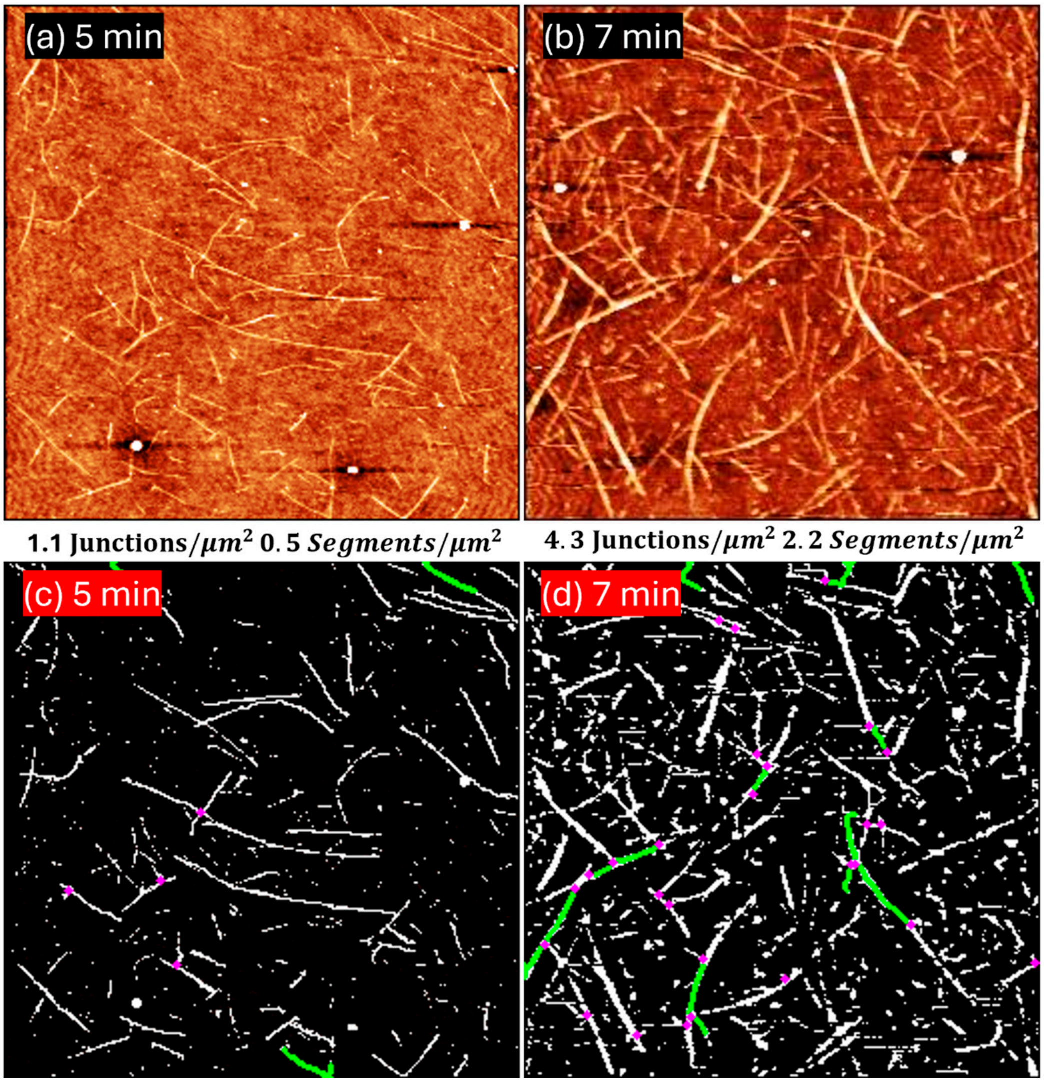

Figure S3: AFM images of the deposited CNT network films of 30% CNT concentration after (a) 5 min and (b) 7 min deposition time. Corresponding binary AFM image overlaid with the analysed CNT network results are shown for 5 min CNT network film in (c) and 7 min CNT network film in (d).

Representative AFM images of CNT films deposited from a 30% CNT dispersion for 10 and 15 min are shown in **Figure S4a** and **b**, and the corresponding quantitative analysis of the CNT networks is presented in **Figure S4c** and **d**. After 10 min of deposition, the CNT network turns into a well-connected structure, exhibiting a significant increase in nanotube coverage and junction density. Extensive interconnected CNT segments are distributed across the analysed region with junction density of $47.5\ \mathrm{Junctions/\mu m^2}$ and segment density of $25.6\ \mathrm{Segments/\mu m^2}$, indicating the formation of multiple conductive pathways. At a CNT deposition time of 15 min, the surface is densely covered by CNTs, resulting in a highly interconnected network that spans the entire analysed area. The substantial increase in CNT coverage leads to a dramatic rise in CNT-CNT junction formation. Unlike the 10 min film, where individual interconnected pathways remain clearly distinguishable, the 15 min film exhibits a uniform and compact morphology with possible nanotube layer overlap. Quantitative analysis of the 30% CNT film deposited for 20 min was not performed because extensive CNT stacking resulted in multiple overlapping layers, making reliable network analysis impractical.

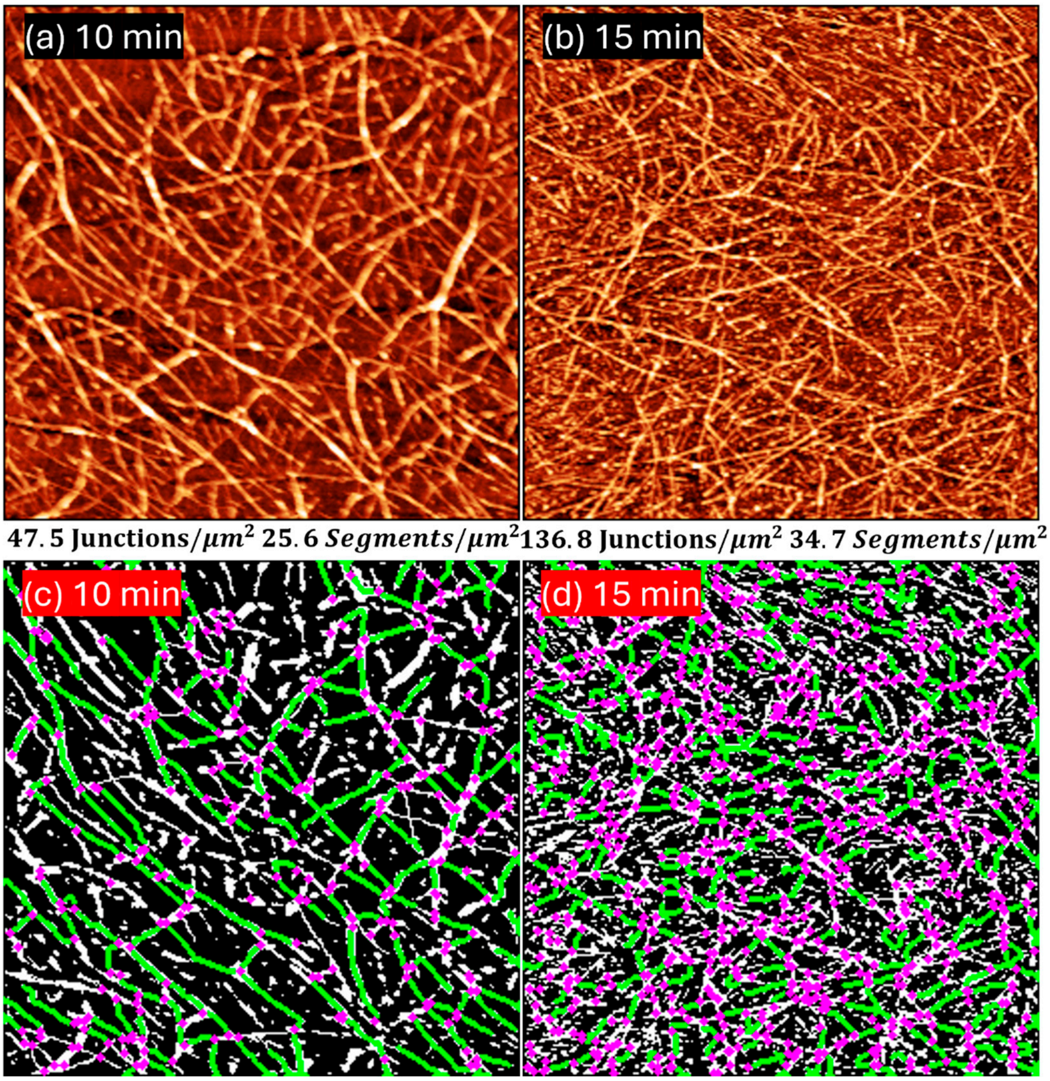

Figure S4: AFM images of the deposited CNT network films of 30% CNT concentration after (a) 10 min and (b) 15 min deposition time. Corresponding binary AFM image overlaid with the analysed CNT network results are shown for 10 min CNT network film in (c) and 15 min CNT network film in (d).

These observations indicate that increasing CNT deposition time promotes progressive network growth, transforming an initially sparse CNT network film into a highly interconnected and dense CNT network film. As all CNT films were fabricated using the same deposition method, a similar trend is expected for the remaining CNT dilutions. This behaviour is further supported by the electrical characterization, where both the I-V and transfer characteristics of the CNT-FET devices show an increase in drain current with increasing deposition time across all CNT dilutions. The increase in current is consistent with the enhanced CNT coverage, higher junction density, and improved network connectivity observed in the morphological analysis.

2. Two-terminal I-V characterization of CNT-FET devices

CNT-FETs with network films fabricated from diluted CNT concentration of 80%, 50%, and 30% of the stock solution were characterised by two-terminal I-V measurements.

Similar trends to those observed for the undiluted (100%) CNT films demonstrated in **Figure 4** of the main manuscript were obtained. Increasing deposition time generally increased the device conductance and produced more linear I-V characteristics, consistent with the formation of denser and more continuous CNT percolation networks.

Figure S5 presents the two-terminal I-V characteristics of devices fabricated using the 80% CNT concentration. The 1-minute deposition produced relatively low current levels and noticeable non-linearity, while the 3 min, 5 min, and 10 min depositions exhibited progressively larger currents and increasingly linear behaviour. In addition, the variation between individual channels decreased with increasing deposition time.

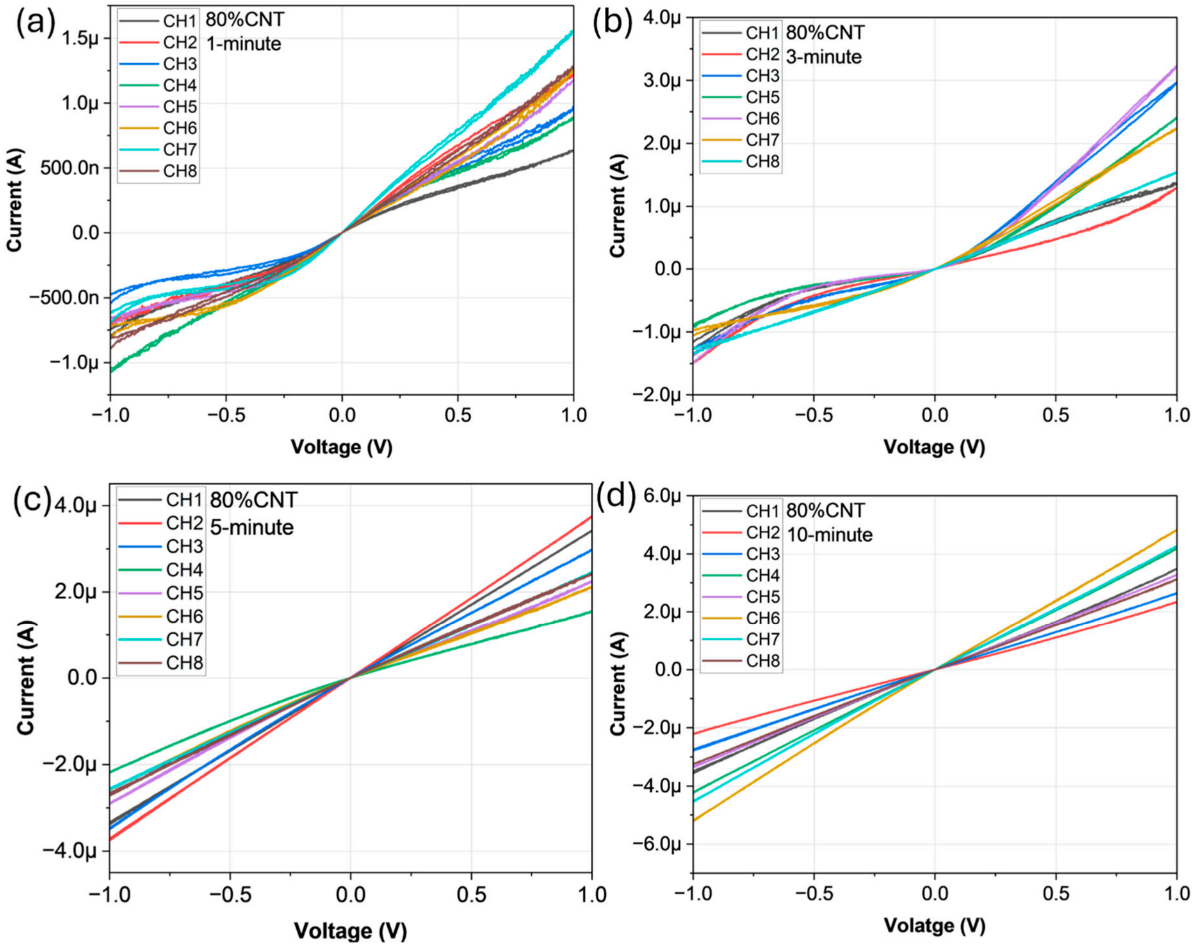

Figure S5: Two-terminal I-V characteristics of CNT Films/Channels measured for devices fabricated with 80% CNT. (a) 1-minute deposition time. (b) 3-minute deposition time. (c) 5-minute deposition time. (d) 10-minute deposition time.

Figure S6 shows the I-V characteristics for devices fabricated using the 50% CNT concentration. Although the same overall trend as for the 100% and 80% CNT solutions

with deposition time was observed, the reduced CNT concentration resulted in lower current levels and more pronounced non-linear characteristics, particularly for the 1 min and 3 min depositions. The 10 min deposition produced the most conductive and reproducible devices within this concentration series.

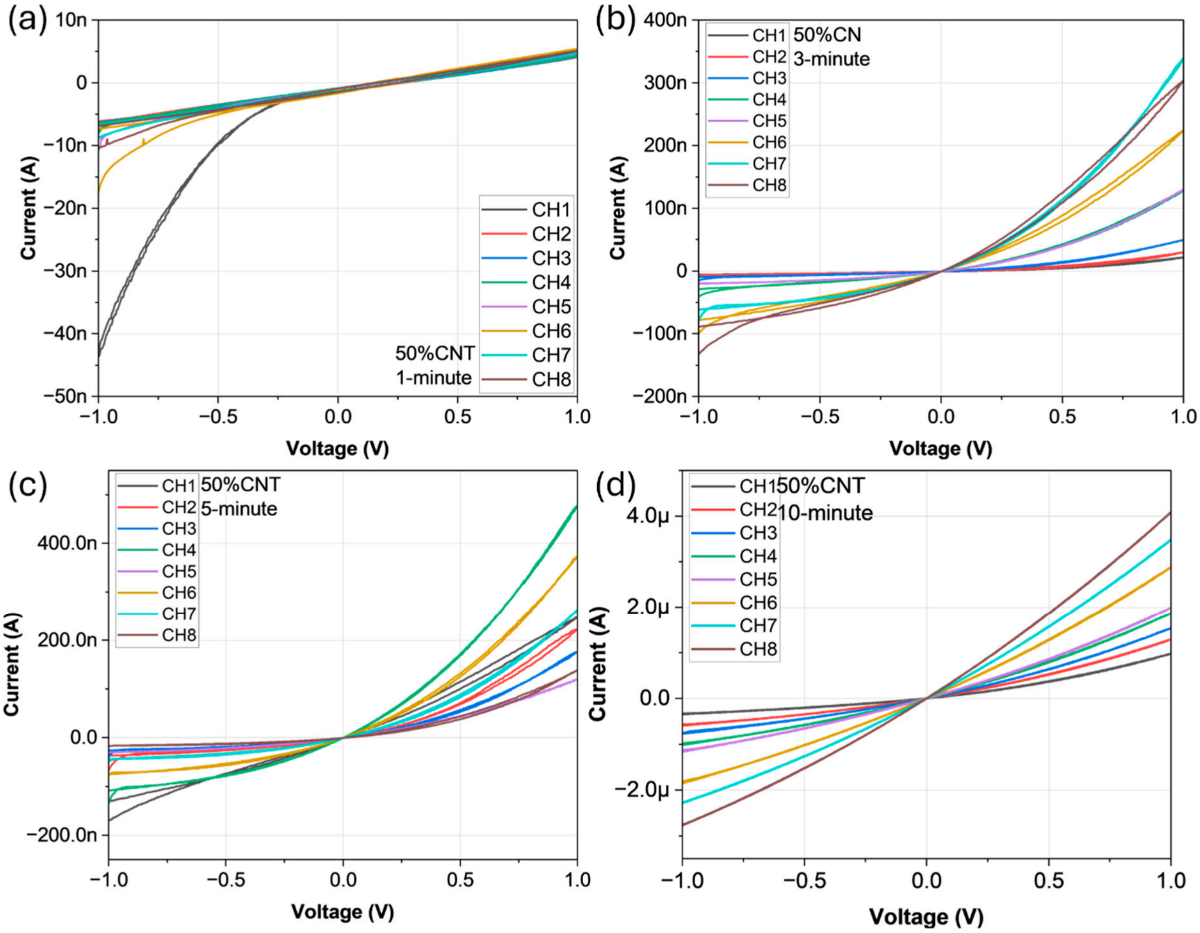

Figure S6: Two-terminal I-V characteristics of CNT Films/Channels measured for devices fabricated with a 50% CNT. (a) 1-minute deposition time. (b) 3-minute deposition time. (c) 5-minute deposition time. (d) 10-minute deposition time.

Figure S7 displays the two-terminal I-V characteristics for devices fabricated using the 30% CNT concentration. These devices exhibited the lowest conductance and the largest variation between individual channels, reflecting the reduced CNT density. Nevertheless, increasing the deposition time from 5 to 20 minutes significantly improved currents and resulted in more linear I-V responses. For the 30% CNT concentration, devices fabricated with the films of under 5-minute deposition time had under percolation CNT network with no conductivity.

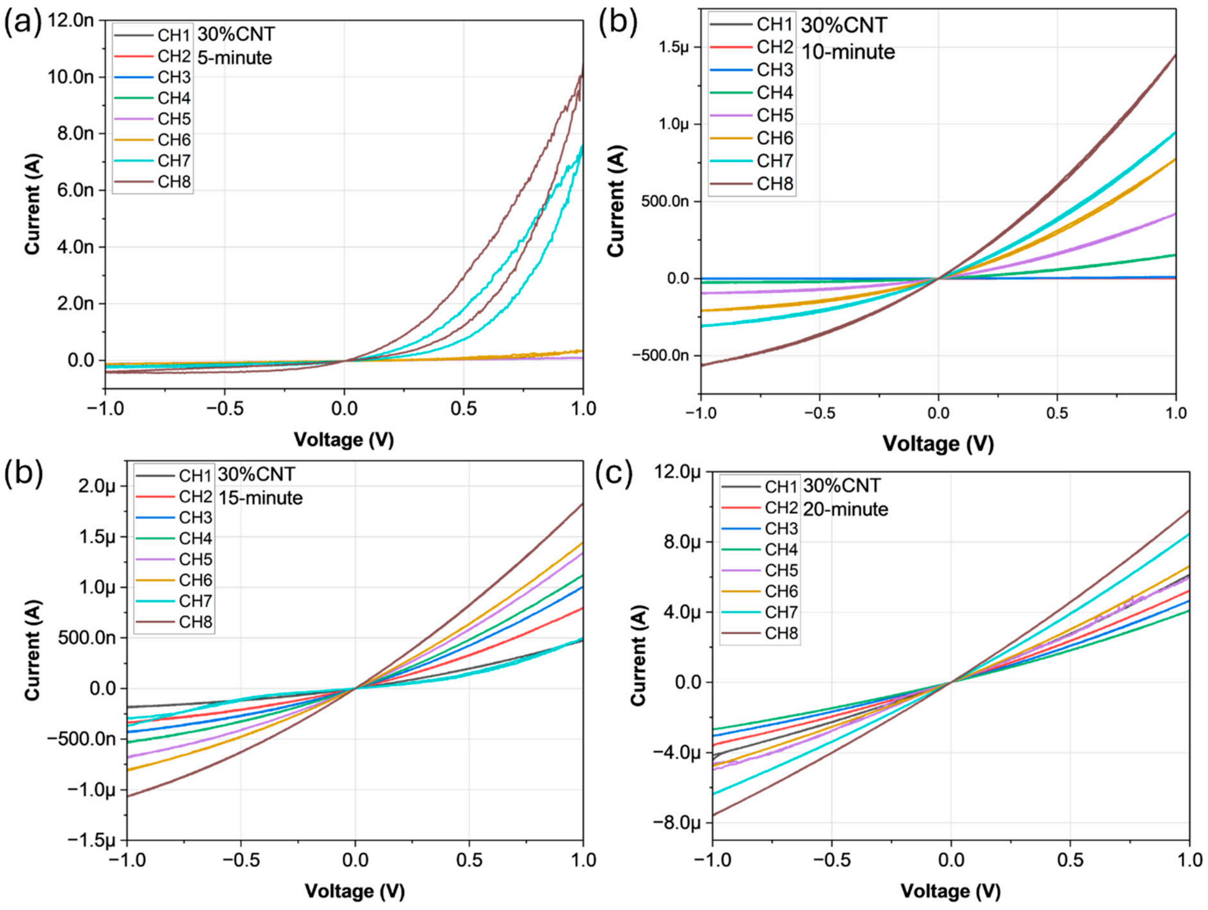

Figure S7: Two-terminal I-V characteristics of CNT Films/Channels measured for devices fabricated with a 30% CNT. (a) 5-minute deposition time. (b) 10-minute deposition time. (c) 15-minute deposition time. (d) 20-minute deposition time.

3. Two-terminal resistance extraction from CNT network I-V characteristics

Figure S8a shows the full I-V curve of the 1-minute CNT network film swept forward and reverse between -1 to 1 V, revealing its non-linear transport behaviour. **Figure S8b** presents a linear fit applied to the selected region of the I-V sweep where the CNT network film exhibited linear behaviour, yielding an excellent fit with an R^2 value of 0.999.

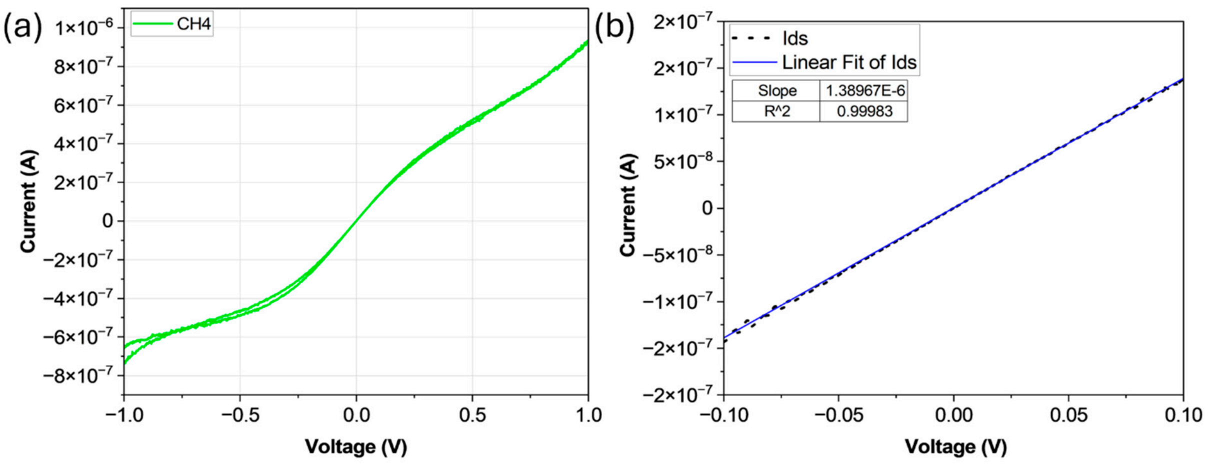

Figure S8: (a) Full I-V characteristics of the 1-minute CNT network film measured from -1 to 1 V, showing non-linear charge transport behaviour. (b) Linear fit applied to the low-bias region of the I-V sweep, demonstrating approximately linear behaviour with a slope of $1.38967{\times}10^{-6}$ and an R^2 value of 0.99983.

The linear region of the current-voltage characteristics shown in **Figure S8b** was fitted using a linear regression, where the slope of linear fit corresponds to the device conductance, $G = \Delta I / \Delta \mathrm{V}$. The total two-terminal resistance (R) of the CNT network is then calculated as the inverse of the conductance, $R = \frac{1}{G}$ and statistically analysed as a function of deposition time for each CNT concentration and deposition time.

4. Liquid-gated transfer characteristics of CNT-FET devices

To investigate the effect of CNT network density on the electrical performance of CNT-FETs operating in a liquid-gated configuration. Transfer characteristics of the devices were measured using the experimental setup described in the characterisation section of the main manuscript. **Figures S9-S11** present the liquid-gated transfer characteristics of CNT-FETs fabricated using diluted CNT concentrations of 80%, 50%, and 30%. For each CNT concentration and each deposition time, three CNT-FET chips containing eight individual CNT-FETs (24 CNT-FETs for each deposition time) were fabricated and characterised. Similar trends to those observed for the undiluted (100%) CNT films discussed in the main manuscript were obtained, with the electrical response strongly dependent on both CNT concentration and deposition time.

Figure S9 presents the liquid-gated transfer characteristics of CNT-FETs fabricated using the 80% CNT concentration. For each device, the gate voltage was swept forward from -1 V to +1 V and subsequently swept in reverse from +1 V to -1 V. Consistent with the

behaviour observed for the undiluted CNT films shown in **Figure 6** of the main manuscript. Increasing the deposition time resulted in higher drain-source currents. The 1 min CNT-FETs exhibited relatively low current level and only 1 functional CNT-FET, while the 3-minute devices showed higher current but with noticeable device-to-device variability. In contrast, the 5 min and 10 min CNT films exhibiting higher current levels, consistent with the formation of denser CNT networks. This trend was also reflected in the device yield. A substantial increase in device yield was observed, with the number of functional individual CNT-FETs increasing from 1 and 3 CNT-FETs for the 1 min and 3 min depositions to 22 and 24 devices for the 5 min and 10 min depositions, respectively.

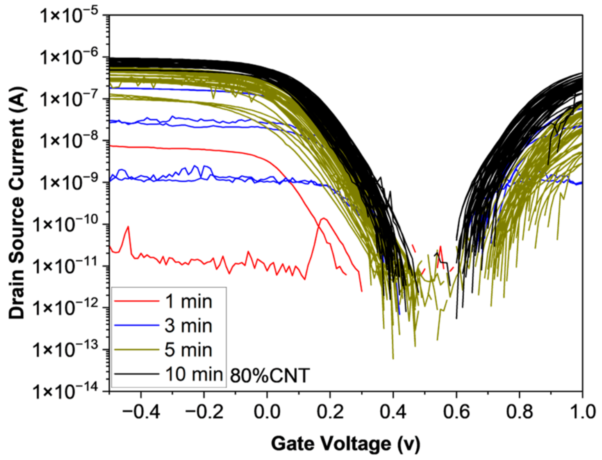

Figure S9: Liquid-gated transfer characteristics of CNT-FET devices fabricated using 80% CNT concentration at deposition times of 1, 3, 5, and 10 min.

Figure S10 shows the liquid-gated transfer characteristics of CNT-FETs fabricated using the 50% CNT concentration. For each device, the gate voltage was swept forward from -1 V to +1 V and subsequently swept in reverse from +1 V to -1 V. No functional devices were obtained for the 1 min deposition, and therefore only the 3, 5, and 10 min transfer curves are presented. As noticed for the higher CNT concentration, longer deposition times generally produced higher drain-source currents, indicating the development of more interconnected CNT networks. The reduced CNT concentration led to increased device-to-device variability and lower overall current levels compared with the 80% CNT devices. The number of working devices for the 1, 3, 5, and 10 min depositions was 0, 13, 8, and 18 channels, respectively.

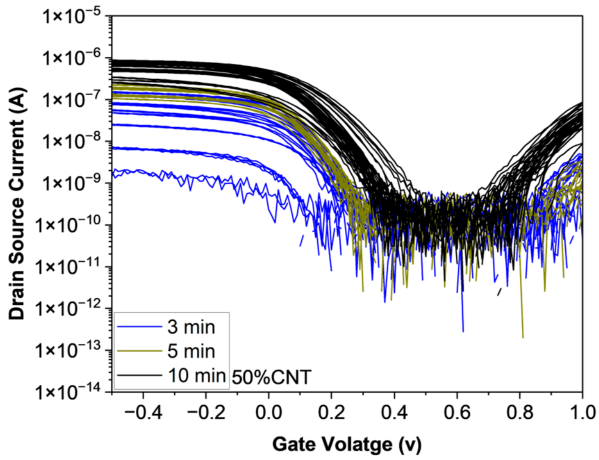

Figure S10: Liquid-gated transfer characteristics of CNT-FET devices fabricated using 50% CNT concentration at deposition times of 3, 5, and 10 min.

Figure S11 shows the liquid-gated transfer characteristics of CNT-FETs fabricated using the 30% CNT concentration. For each device, transfer characteristics were recorded during both forward sweep from -1 V to +1 V and reverse sweep from +1 V to -1 V. Deposition times shorter than 5 min were not investigated, as they produced under-percolated CNT networks. Consequently, only the 10, 15, and 20 min depositions are presented. Despite the lower CNT concentration, extending the deposition time progressively improved the conductance of the CNT network, with the longer depositions exhibiting increased drain-source currents. The functional channel yield reflected this trend, with 0, 5, 5, and 23 devices exhibiting functional liquid-gated behaviour for the 5, 10, 15, and 20 min depositions, respectively.

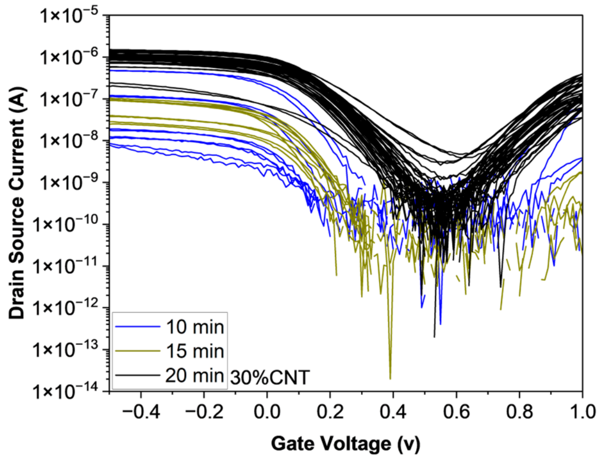

Figure S11: Liquid-gated transfer characteristics of CNT-FET devices fabricated using 30% CNT concentration at deposition times of 10, 15, and 20 min.

5. References

1. Begzaad, sangar; Happe, E.D.; Gouws, G.; Plank, N. AFM Analysis of Morphology–Density–Transport Relationships in Carbon Nanotube Thin Films. *Nanotechnology* **2026**.
